# Sustainable Valorization of *Posidonia* Waste Ash for Phosphate Removal: A Surface Complexation Approach Under Variable Water Chemistry

**DOI:** 10.3390/molecules30173639

**Published:** 2025-09-06

**Authors:** Jesús Mengual, Juan A. González

**Affiliations:** 1 Instituto de Tecnología Química, Universitat Politècnica de València—Consejo Superior de Investigaciones Científicas, Avenida de los Naranjos s/n, 46022 València, Spain; 2 Institut Universitari d’Investigaciò d’Enginyeria de l’Aigua i Medi Ambient (IIAMA), Universitat Politècnica de València, Camí de Vera s/n, 46022 Valencia, Spain; juagonr1@hma.upv.es

**Keywords:** phosphate removal, *Posidonia oceanica* ash, surface complexation modeling, ternary complexes, phosphate–carbonate competition, non-electrostatic model, variable chemical conditions

## Abstract

Phosphorus pollution represents a persistent and significant threat to aquatic ecosystems, particularly within the Mediterranean region, where ongoing eutrophication continues to compromise both water quality and biodiversity. Concurrently, the accumulation of *Posidonia oceanica* residues along coastal areas presents a biomass management challenge. This study explores the sustainable use of thermally treated *Posidonia* ash as a low-cost, bio-based adsorbent for phosphate removal from water. Batch experiments under varying phosphate concentrations, pH, hardness, and alkalinity revealed high removal capacities (33.5–58.7 mg/g). A novel surface complexation model (SCM) was developed and validated using spectroscopic techniques to elucidate the mechanisms of phosphate retention. The SCM outperformed conventional isotherm models by providing mechanistic insights into adsorption behavior. Phosphate adsorption was found to be pH-dependent, occurring via surface complexation to neutral and basic surface sites. The release of Ca^2+^ and Mg^2+^ ions facilitated ternary complex formation and precipitation. Under alkaline conditions, competitive adsorption between phosphate and carbonate ions was observed. This study demonstrates the dual benefit of *Posidonia oceanica* ash: efficient phosphate removal and its reuse as a phosphorus reservoir, offering a circular strategy for tackling nutrient pollution and promoting coastal biomass valorization.

## 1. Introduction

Increasing anthropogenic pressures on aquatic ecosystems have led to a marked rise in phosphorus pollution across European water bodies [1]. Although phosphorus is vital for biological productivity, its excess in aquatic environments serves as a pollutant, driving oxygen depletion and biodiversity loss through algal blooms in eutrophic systems [2]. This problem is especially pronounced in semi-enclosed basins such as the Mediterranean Sea, where restricted water exchange and elevated nutrient inputs from agriculture and urban runoff intensify eutrophication processes [3].

Simultaneously, Mediterranean coastal zones are experiencing an increasing accumulation of *Posidonia oceanica* residues. Although this endemic seagrass plays a vital ecological role in sediment stabilization and supporting marine biodiversity [4], climate change-induced stressors, such as rising sea temperatures and altered hydrodynamics, have intensified the deposition of *Posidonia* biomass along shorelines [5]. Despite their high organic and mineral content, these residues are often treated as waste, overlooking their potential for valorization [6].

Recent studies have explored the thermal treatment of *Posidonia oceanica* biomass to produce ash materials with potential adsorptive properties [7]. Ashes rich in mineral phases have shown promising capabilities for phosphate retention from aqueous solutions [8,9], and their application as sustainable adsorbents aligns with circular economy principles. Consequently, repurposing *Posidonia* litter as a precursor for adsorbent materials offers dual environmental benefits: the valorization of coastal biomass and the mitigation of phosphorus pollution in aquatic systems.

Traditionally, phosphate adsorption behavior has been described using empirical isotherm models such as Langmuir and Freundlich. However, these models are limited in capturing the dynamic interactions occurring under variable chemical conditions in natural waters, particularly due to their inability to account for synergistic and competitive effects among multiple components [10]. As a result, their predictive capacity is significantly limited in real-world scenarios.

In contrast, thermodynamic models offer a powerful alternative [11], as they can incorporate surface interactions through surface complexation models (SCMs). Unlike empirical models, SCMs integrate thermodynamic principles and chemical speciation to describe the formation of specific surface complexes between adsorbates and reactive surface sites [12]. This approach enables a more mechanistic and transferable understanding of the adsorption phenomena, especially under variable environmental conditions. Nevertheless, despite their advantages, SCMs remain underutilized in the context of phosphate adsorption, because of their higher complexity.

To overcome these limitations, this study introduces a novel application of SCMs to describe phosphate retention on *Posidonia oceanica* ash. By integrating thermodynamic modeling with advanced instrumental techniques, we aim to identify the surface species formed during phosphate adsorption under varying chemical conditions and quantify their relative contributions. This combined approach enables a detailed elucidation of retention mechanisms and provides a robust framework for predicting adsorbent performance across diverse water chemical conditions.

Particular attention is given to the roles of sodium, magnesium, calcium, and carbonate ions, common constituents of Mediterranean waters, due to their known interactions with phosphate [13]. Calcium, for instance, can enhance phosphate retention via precipitation or surface complexation, whereas carbonate species may compete for adsorption sites or alter the adsorbent’s surface charge. The synergistic or antagonistic effects of these ions remain largely unexplored in the context of novel biosorbents such as *Posidonia* ash [14].

Beyond water treatment applications, the reuse of phosphate-saturated *Posidonia* ash as a soil amendment presents a promising strategy for nutrient recovery and sustainable agriculture. The agronomic value of the spent adsorbent depends on the chemical form of the retained phosphorus, which governs its solubility and bioavailability [15]. By characterizing the surface-bound phosphate species, we aim to assess the potential of this material as a reservoir for phosphorus and contribute to the development of integrated nutrient management strategies.

This study addresses a pressing environmental challenge through the innovative application of surface complexation modeling to a novel marine biomass-derived adsorbent. The proposed SCM framework captures the influence of pH, ionic strength, water hardness, and alkalinity, offering improved predictive capabilities over traditional models. By identifying specific surface species, the model provides insights into the stability, reversibility, and dynamics of phosphate binding. The integration of modeling and spectroscopy enables a comprehensive understanding of phosphate retention mechanisms and supports the development of sustainable solutions for water remediation and resource recovery.

The main objectives of this study are to

- Develop and characterize a novel adsorbent material derived from *Posidonia oceanica* residues for phosphorus removal.
- Establish a thermodynamic modeling approach supported by instrumental techniques.
- Elucidate the dominant phosphate retention mechanisms under variable chemical conditions.
- Validate model predictions and analyze the sensitivity of its parameters.
- Evaluate the potential reuse of the spent material for nutrient recovery via gradual phosphorus release.

The findings are expected to have broad implications in the fields of environmental chemistry, materials science, and applied chemistry, particularly within the frameworks of the circular economy and coastal ecosystem management.

## 2. Results and Discussion

### 2.1. Adsorbent Characterization

#### 2.1.1. Surface Area and Porosity

To assess the physicochemical properties of the newly developed adsorbent, surface area and porosity analyses were performed. As summarized in Table 1, the *Posidonia oceanica* ash exhibited a BET surface area of 13.25 m^2^/g, with approximately 86% of the total surface area attributed to external surfaces. The micropore volume was relatively low (0.84 cm^3^/kg), indicating that the material is predominantly mesoporous to macroporous in nature. Although this surface area is significantly lower than that of activated carbons or engineered biochars, it may still be suitable for applications such as soil amendment, where high surface area and low microporous structure are not a critical requirement.

The ash also exhibited an alkaline point of zero charge (pH_PZC_) of 9.1 at a solid-to-liquid ratio of 0.1 g/L, suggesting a high density of protonable surface functional groups. This property enhances its potential for neutralizing acidic environments, such as wastewater or acidic soils. Notably, the pH_PZC_ increased progressively with higher solid-to-liquid ratios, reaching 9.6, 10.5, and 10.9 at 0.5, 1, and 2 g/L, respectively. This trend is consistent with observations reported for similar ash-based adsorbents [16].

#### 2.1.2. Elemental and Chemical Composition

Elemental analysis (Table 1) revealed a low carbon content (3.42%), along with negligible nitrogen (0.01%), low hydrogen (0.48%), and moderate sulfur (1.85%) levels. These values suggest a high degree of combustion and minimal residual organic matter, consistent with the characteristics of thermally treated marine biomass.

The chemical composition of the ash indicates a high concentration of silica (SiO_2_, 23.64%) and calcium oxide (CaO, 14.48%), accompanied by notable amounts of magnesium oxide (MgO, 8.44%) and iron oxide (Fe_2_O_3_, 5.31%). Minor constituents include alumina (Al_2_O_3_, 2.67%), sodium oxide (Na_2_O, 1.62%), and potassium oxide (K_2_O, 0.89%), which are present in residual quantities. Although the phosphorus content (P_2_O_5_, 0.39%) is relatively low, it may still influence phosphate removal through leaching mechanisms under specific environmental conditions. These compositional features are consistent with those reported for other marine macroalgae [17] and seaweed-derived [18] ashes.

#### 2.1.3. Thermal Stability, Morphology, and Crystalline Structure

**Figure 1:**
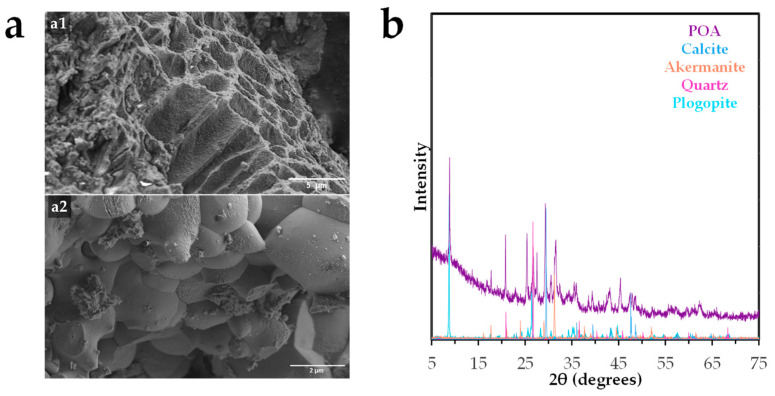
Morphological, and structural characterization of *Posidonia oceanica* ash: (**a**) Scanning electron microscopy (SEM) images of (**a1**) tubular channels and (**a2**) granular texture, and (**b**) X-ray diffraction (XRD) pattern.

Figure 1 presents the results of the various analyses performed to elucidate the morphology and structural characteristics of the synthesized adsorbent surface.

Scanning electron microscopy (SEM) images (Figure 1a) reveal a heterogeneous and porous microstructure. As shown in Figure 1(a1), the surface exhibits a rough, stratified morphology with irregular tubular channels ranging from 2 to 4 µm in diameter, likely formed by the collapse and sintering of organic matrices during thermal treatment. These structural features contribute to increased microporosity and may enhance the material’s reactivity by providing additional active sites. Figure 1(a2) displays a granular texture composed of angular crystalline particles (1–2 µm in diameter) embedded within the ash matrix. These particles are likely the result of recrystallization of inorganic components such as silicates and carbonates at high temperatures. The presence of cavities and voids further supports the potential of this material for applications involving adsorption, ion exchange, or soil conditioning. Similar morphologies have been reported in ashes derived from other vegetal wastes [19].

The X-ray diffraction (XRD) pattern (Figure 1b) confirms the presence of a certain crystalline structure, with distinct peaks corresponding to quartz (SiO_2_), calcite (CaCO_3_), and calcium/magnesium-rich silicates as akermanite (Ca_2_MgSi_2_O_7_), phyllosilicates as phlogopite (KMg_3_(AlSi_3_O_10_)(OH)_2_), iron oxides as hematite (Fe_2_O_3_), and minor phases such as sodium or potassium aluminosilicates. These mineral phases are consistent with the chemical composition previously determined and suggest potential pozzolanic reactivity, enabling its future use in cementitious materials or geopolymer synthesis. Comparable mineralogical profiles have been observed in ashes derived from marine algae [20].

#### 2.1.4. Surface Charge Behavior of the Adsorbent

**Figure 2:**
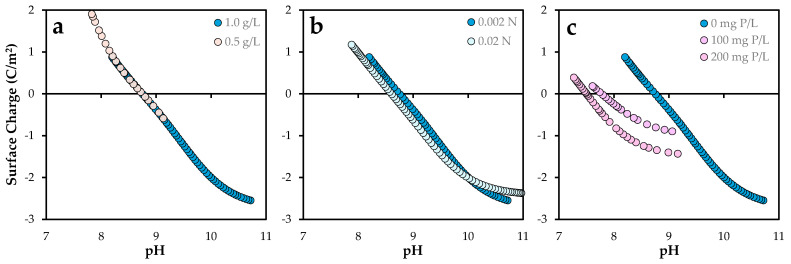
Surface charge of *Posidonia oceanica* ash: Effect of (**a**) solid–liquid ratio: 0.5–1.0 g/L, (**b**) ionic strength: 0.02–0.002 N, and (**c**) initial phosphorus load: 0–200 mg P/L. The standard error of the mean ranged from 0.02 to 0.07 C/m^2^.

The acid–base behavior of the adsorbent surface was evaluated by examining its surface charge as a function of pH under varying experimental conditions, including adsorbent dose, ionic strength, and phosphorus content following prior adsorption. The experimental conditions are detailed in Figure 2.

Potentiometric acid–base titrations (Figure S1) were conducted on aqueous suspensions of *Posidonia oceanica* ash to determine the surface charge. Figure 2 presents the evolution of surface charge with pH under the different experimental conditions.

As shown in Figure 2a, the surface charge profiles for doses of 0.5 and 1.0 g/L follow a continuous trend, indicating that the intrinsic acid–base properties of the surface functional groups remain largely unaffected by the solid concentration. The point of zero charge (pH_PZC_) remained relatively constant at approximately 8.75. The magnitude of the surface charge suggests a high density of amphoteric reactive sites, consistent with previous studies on bio-based adsorbents [21]. These values are slightly lower than those obtained using the solid-addition method. In this approach, the point of zero charge (PZC) is defined as the pH at which ΔpH (pH_final_–pH_initial_) equals zero. As previously noted, ΔpH values for *Posidonia oceanica* ash (POA) were found to vary with the solid-to-liquid ratio. This observation suggests that fixed surface charges and mineral dissolution processes may influence the final pH, particularly at higher S/L ratios. In contrast, the potentiometric titration method involves shorter contact times, which minimizes these effects and yields more consistent pHPZC values. This methodological distinction accounts for the observed differences between the two techniques (see Figure S2).

In contrast, increasing the ionic strength from 0.002 N to 0.02 N (Figure 2b) resulted in a slight reduction in positive surface charge at lower pH values, accompanied by a minor shift in pH_PZC_ to 8.62, as observed for other solids [22]. This behavior may be attributed to competitive interactions between protons and background electrolyte cations (Na^+^) for surface sites, as well as compression of the electrical double layer, in accordance with the Gouy–Chapman–Stern model [23].

Phosphorus pre-adsorption had a pronounced effect on the surface charge profile (Figure 2c). As the phosphorus content increased, the positive surface charge at low pH values decreased progressively, as well as the pH_PZC_ values (7.5–7.8). This trend suggests that phosphate species occupy or modify proton-active surface sites, likely forming inner-sphere complexes that alter the surface’s acid–base behavior [24]. These modifications in surface speciation and site saturation are critical for accurately modeling adsorption mechanisms and optimizing operational conditions for contaminant removal [25].

### 2.2. Phosphate Removal Performance

The total phosphate removal capacity of *Posidonia oceanica* ash was systematically evaluated under a range of water chemistry conditions, including variations in phosphate concentration, adsorbent dosage, pH, alkalinity, and water hardness (Table S1). Phosphate uptake by POA varied significantly depending on the specific water chemistry, with removal capacities ranging from 1.08 to 1.89 mmol/g (33.5–58.7 mg/g). Under conditions favoring surface precipitation, POA achieved removal capacities up to 2.8 mmol/g. Table 2 provides a comparative overview of phosphate removal capacities reported in the literature for various bio-based adsorbents. As shown in Table 2, its performance surpasses that of other bio-based adsorbents, including ashes, biochars, and activated carbons from residual biomass [13,26], highlighting its strong potential for efficient phosphorus recovery from aqueous environments.

**Figure 3:**
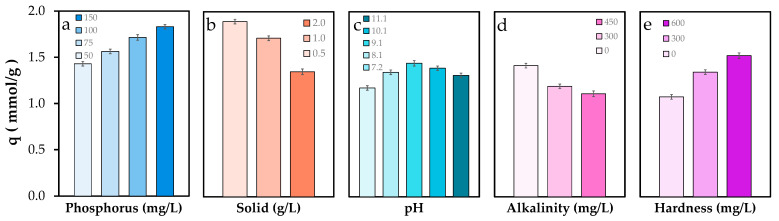
Total phosphate removal capacity of POA under variable water chemistry conditions: (**a**) Phosphate concentration, (**b**) solid dose, (**c**) pH, (**d**) alkalinity, and (**e**) water hardness. Alkalinity and water hardness are referred to as CaCO_3_. Error bars represent the standard error of the mean.

Figure 3 illustrates the total phosphate removal behavior of POA under different chemical conditions. As shown in Figure 3a, increasing the initial phosphate concentration led to a progressive enhancement in removal capacity, from 1.43 to 1.83 mmol/g. This trend aligns with classical adsorption theory, where higher solute concentrations increase the mass transfer driving force, thereby promoting greater phosphate uptake [43]. Conversely, increasing the adsorbent dose from 0.5 to 2.0 g/L resulted in a 29% decrease in removal capacity (from 1.89 to 1.35 mmol/g; Figure 3b). This reduction may be attributed to the concomitant increase in solution pH at higher dosages [43]. Indeed, total phosphate removal exhibited a bell-shaped dependence on pH (Figure 3c), with a maximum capacity of 1.44 mmol/g at pH 9. Removal efficiency declined by 18% and 10% at pH 7 and 11, respectively, likely due to changes in phosphate speciation (from H_2_PO_4_^−^ to HPO_4_^2−^ and PO_4_^3−^), which influence binding affinity.

Increasing alkalinity led to a marked decline in phosphate removal, from 1.42 to 1.11 mmol/g (Figure 3d), representing a 21% reduction. This effect may be due to competitive adsorption between carbonate species and phosphate ions [44]. Additionally, changes in buffering capacity could also alter phosphate speciation and surface binding dynamics [45]. Finally, Figure 3e shows a notable increase in phosphate removal capacity, from 1.08 to 1.53 mmol/g, under conditions of elevated water hardness. This enhancement is likely due to the formation of ternary surface complexes involving calcium or magnesium ions [46], which can act as bridges between phosphate and the adsorbent surface or even precipitate as mineral phases, thereby contributing to improved phosphate removal.

### 2.3. Surface Complexation Modeling

#### 2.3.1. Approach and Implementation

The variability in phosphate adsorption capacity under different chemical conditions poses challenges for the application of traditional isotherm models (e.g., Langmuir, Freundlich), which are widely used in this field. In contrast, surface complexation models (SCMs) provide a more robust framework for simulating variable chemical environments through thermodynamic formulations that require the identification of surface speciation of the adsorbate. Among available SCMs, the non-electrostatic model (NEM) was selected in this study due to its mathematical simplicity and proven ability to represent surface interactions effectively [47].

While NEM has been applied in some studies to describe the acid–base behavior of adsorbents [48] and phosphate adsorption mechanisms [49], its use remains less widespread than other SCMs such as the constant capacitance model (CCM), diffuse layer model (DLM), triple layer model (TLM), or charge distribution model (CD-MUSIC), which typically involve greater computational complexity. Nevertheless, Arora et al. [47] highlighted the potential of NEM to support performance assessment models, particularly due to its computational efficiency, making it a promising alternative for broader application.

Visual MINTEQ was employed as the simulation platform. This chemical equilibrium model enables the calculation of inorganic speciation, solubility equilibria, and adsorption processes in natural waters. Although originally developed for geochemical equilibrium systems, Visual MINTEQ is also capable of simulating a wide range of chemical processes, including dissolution–precipitation, redox reactions, and adsorption. The model relies on well-established thermodynamic data to define chemical species.

**Figure 4:**
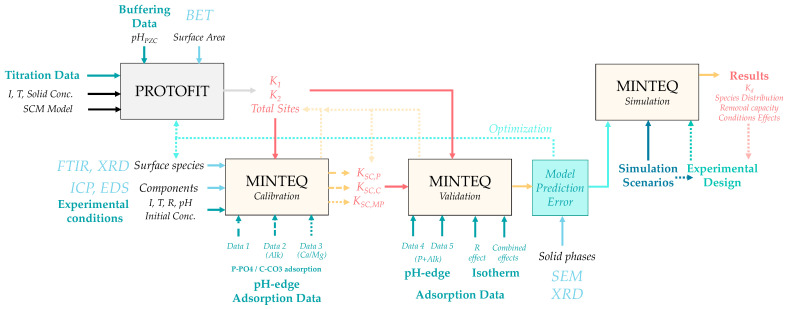
Workflow of the modeling methodology defined in the study.

To identify the relevant model components, 10 g/L of POA was contacted with deionized water for five days. The resulting solutions were analyzed by ICP under varying pH conditions (Table S2). The analysis revealed the release of Ca, Mg, Na, K, and Si from POA, while other elements present in the solid matrix (shown in Table 1) were not detected in solution. The concentrations of these elements decreased with increasing pH. Based on these findings, the aqueous components selected for the model included H^+^, H_2_O, Ca^2+^, Mg^2+^, K^+^, Na^+^, H_4_SiO_4_, phosphate, and carbonate, the latter two being the primary adsorbates under study. Iron and aluminum were excluded due to their absence in solution. Chloride, although present as a counterion in some experimental salts, was not found to significantly influence the results. Table 3 lists the 34 aqueous species considered in the model, along with gaseous CO_2_. Table S3 presents the 36 potential mineral phases derived from the selected components. A schematic overview of the modeling procedure is provided in Figure 4.

First, potentiometric titration data (Figure S1) were analyzed using the Protofit 2.1 software [50] to determine the concentration of surface sites and the (de)protonation constants of amphoteric groups. Next, surface species were defined based on the results of phosphate adsorption experiments under variable conditions, supported by solid-phase spectroscopic analyses. The equilibrium constants of the proposed surface complexes were optimized using Visual MINTEQ, based on experimental pH-edge data for phosphate removal. These constants were subsequently validated through simulations using independent experimental datasets. Error estimation from this phase, together with surface characterization data, was used to confirm or refine the proposed surface speciation.

Some studies employ a large number of surface species resulting from the interaction between phosphate and active surface sites [51], or combinations of different types of surface groups [52]. However, in the present study, efforts were made to implement the minimum number of interactions between the adsorbate and the solid surface, thereby reducing the number of independently adjustable parameters.

Table 4 summarizes the surface species included in the model along with their stoichiometry. The formation of acidic and basic species through protonation and deprotonation of surface sites was first considered. A stable phosphate surface complexes was incorporated to reflect the pH-dependent adsorption behavior of POA. Additionally, based on the observed effects of Ca^2+^, Mg^2+^ and Na^+^ ternary complexes involving phosphate and these cations were proposed, acting as bridging ions that facilitate phosphate interaction with basic surface sites [53]. Finally, the experimentally observed decrease in phosphate retention under high carbonate concentrations, particularly in alkaline conditions, was attributed to competitive adsorption of carbonate, which was also incorporated into the model.

#### 2.3.2. Parameter Determination

Active site concentration and the constants K_1_ and K_2_ were initially determined independently through acid–base titration of the adsorbent. These preliminary estimations were obtained using the Protofit 2.1 software and are presented in Table 5.

Subsequently, slight adjustments were made following simulations conducted with Visual MINTEQ. The final values used throughout the study are shown in Table 4. The active site concentration was set at 1.5 mM (equivalent to 0.0015 mol/g), which is approximately 10% lower than the value estimated by Protofit (0.0017 mol/g). This reduction may be attributed to the limited contribution of the microporous structure of the adsorbent, which accounts for only 14% of the total surface area. The final values for log K_1_ and log K_2_ were 7.7 and –8.9, respectively, with the latter slightly adjusted from the Protofit-derived value of –9.3.

The obtained values for K_1_ and K_2_ are consistent with those reported for iron [54,55] and aluminum oxides [56], suggesting that the active sites in POA may be associated with Fe/Al-based structures.

**Figure 5:**
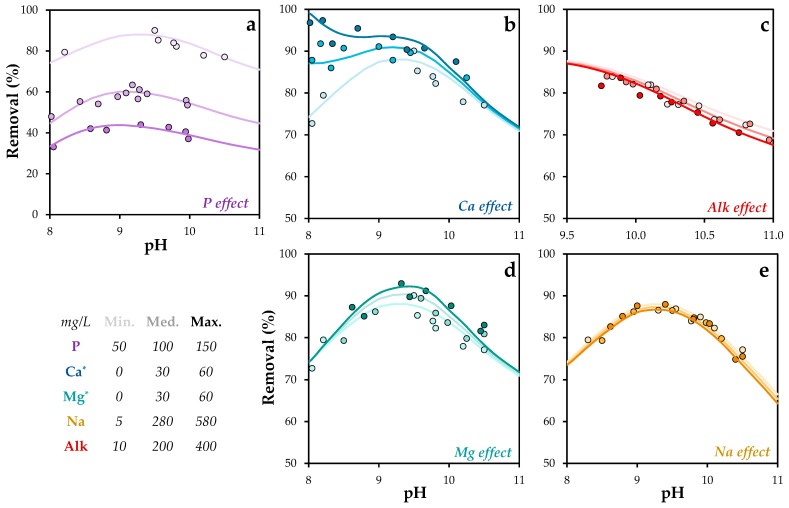
Experimental and simulated phosphorus removal as a function of pH under different chemical conditions: Effect of (**a**) phosphorus, (**b**) calcium, (**c**) alkalinity, (**d**) magnesium, and (**e**) sodium concentrations. P = 1.6 mM. T = 293 K. The standard error of the mean ranged from 0.9 to 1.8%. * It refers to concentration added to water.

The equilibrium constants for the remaining surface complexes were individually fitted based on experimental assays conducted under varying chemical conditions. Figure 5 presents both the experimental data and the model simulations based on the proposed surface complexation model. The corresponding equilibrium constant values are listed in Table 4.

A contact time of 96 h was considered sufficient to ensure chemical equilibrium. As shown in Figure S3, the system progressively approached equilibrium, with differences between measurements decreasing to 3–5% after 72 h. Beyond 96 h, the variations consistently fell below 1–2%, indicating that a stable state was achieved under all tested chemical conditions.

Phosphorus removal efficiency using POA was strongly influenced by pH and chemical composition. Experimental results showed that removal increased with higher phosphorus concentrations. Optimal removal was observed at pH values between 9 and 10. Elevated calcium concentrations enhanced phosphorus removal, likely due to the formation of insoluble precipitates, consistent with previous findings [57,58,59].

The formation of ternary complexes involving anions and metal elements is a well-documented mechanism that promotes solid–adsorbate interactions [60]. Zhao et al. [61] identified the formation of ternary phosphate–copper complexes as a key mechanism in phosphate retention on mineral surfaces. In this context, the observed increase in phosphate removal in the presence of magnesium may be attributed to the formation of similar ternary surface complexes.

In contrast, sodium exhibited only a minor effect on phosphorus removal, despite a significant increase in its concentration, indicating a limited role in phosphate retention mechanisms. Alkalinity had a moderate impact, with a slight decrease in removal capacity, likely due to competitive interactions between phosphate and carbonate anions for active sites [62].

Although the specific surface species considered in this model have not been previously reported in the literature, Tang et al. [63] reported higher log K values for phosphate species interacting with Fe (12.0) and Al oxides (13.6). These values suggest a stronger affinity than that observed for POA, indicating that the active sites in POA may differ partially in nature from those of Fe/Al oxides.

Overall, the experimental findings support the use of POA in combination with pH control and targeted chemical dosing as an effective strategy for optimizing phosphorus removal in wastewater treatment applications.

#### 2.3.3. Phosphorus Speciation and Removal Efficiency

**Figure 6:**
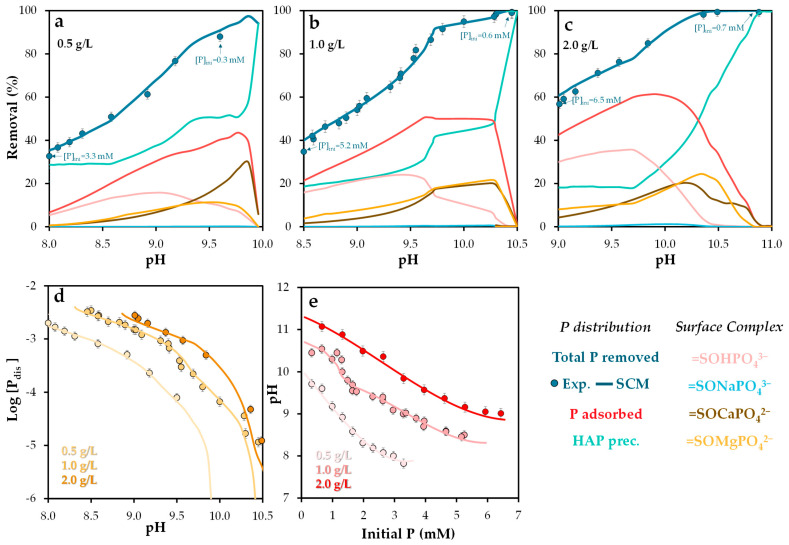
Experimental and Visual MINTEQ-simulated phosphorus removal and surface phosphorus speciation as a function of pH, under varying initial phosphorus concentrations (5–200 mg/L), at solid doses of (**a**) 0.5, (**b**) 1.0, and (**c**) 2.0 g/L; (**d**) experimental and simulated equilibrium dissolved phosphorus as a function of pH; and (**e**) experimental and simulated pH as a function of initial phosphorus concentration. Error bars represent the standard error of the mean.

In order to evaluate the effect of pH and POA dosage on phosphorus removal efficiency, experiments were conducted using different initial phosphate-to-POA ratios. Figure 6 presents both experimental and Visual MINTEQ-simulated results for phosphorus removal efficiency under varying doses of POA (0.5, 1.0, and 2.0 g/L) across a pH range of 8–11. Variations in adsorbate and adsorbent concentrations significantly influenced removal efficiency. The results show a clear enhancement in phosphorus removal with increasing POA dosage, particularly at higher pH values (see experimental isotherm in Figure S4a). This improvement is likely associated with the greater availability of active sites. Additionally, higher POA doses resulted in elevated final pH values for solutions with similar initial conditions, due to the buffering capacity of the adsorbent and its high point of zero charge (pH_PZC_).

At a dose of 0.5 g/L (Figure 6a), phosphorus removal via precipitation of insoluble solid phases exceeded that achieved through adsorption. However, this trend reversed at 1.0 g/L (Figure 6b) and 2.0 g/L (Figure 6c), where adsorption became the dominant mechanism, except at pH values above 10.5, where precipitation again prevailed [64]. Simulations indicated that the solid phase hydroxyapatite (Ca_5_(PO_4_)_3_(OH)) exceeded its saturation index across the entire pH range studied, suggesting partial phosphorus removal via precipitation, driven by calcium presence and alkaline conditions. These findings are consistent with previous studies [51,52], which highlight the role of hydroxyapatite formation in phosphorus retention.

Regarding adsorption, the dominant surface complex was =SOHPO_4_^3−^ at pH values below 9.3 (0.5 g/L), 9.7 (1.0 g/L), and 10.2 (2.0 g/L). Above these thresholds, ternary complexes such as =SOMgPO_4_^2−^ and =SOCaPO_4_^2−^ became predominant [62], particularly within the pH range of 9.5–10.5. Atouei et al. [65] reported that phosphate adsorption is significantly higher in Ca–PO_4_ systems than in Mg–PO_4_ systems, which aligns with the present findings.

In contrast, the formation of =SONaPO_4_^3−^ was negligible, indicating its limited relevance as a retention mechanism. Atouei et al. [65] also noted substantial differences in adsorption energies between Ca/Mg and Na, likely due to distinct adsorption mechanisms. This supports the conclusion that variations in ionic strength have minimal impact on phosphorus retention.

The simulation results closely matched the experimental data under all conditions, including dissolved phosphorus and pH values (Figure 6d,e), validating the predictive capability of the NEM surface complexation model employed.

**Figure 7:**
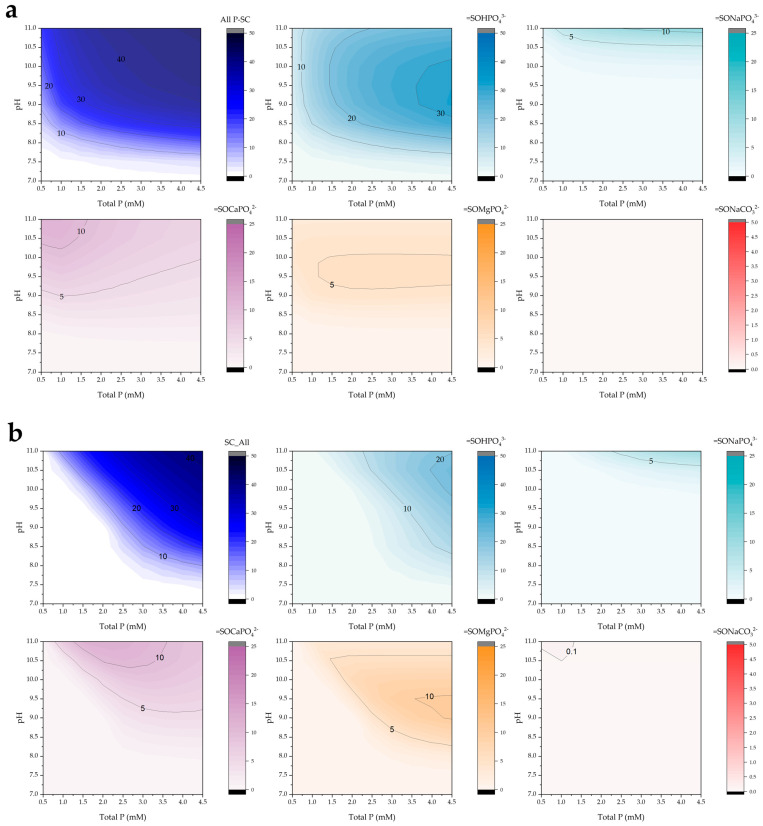
Simulated surface complex predominance (mg/g) as a function of pH and total phosphorus under low alkalinity conditions (10 mg/L) for POA doses of (**a**) 0.5 and (**b**) 2.0 g/L.

The surface speciation diagrams presented in Figure 7 illustrate the distribution of phosphate and carbonate complexes formed on the POA adsorbent as a function of pH and total phosphorus concentration. The total surface-bound phosphorus species (all P-SC) exhibit a clear increase in surface coverage with rising phosphorus concentrations, particularly under alkaline conditions (pH > 8).

Among the phosphate species, the =SOHPO_4_^3−^ complex is predominant across a wide pH range, especially at higher phosphorus concentrations, with maximum surface concentrations observed between pH 9 and 10. A similar pH-dependent trend is observed for the =SOMgPO_4_^2−^ complex, although its overall abundance is significantly lower, possibly due to competitive interactions or the lower affinity of magnesium for the surface.

The formation of ternary complexes involving sodium and calcium, such as =SONaPO_4_^3−^ and =SOCaPO_4_^2−^, becomes more pronounced at elevated pH values, suggesting their increasing relevance under alkaline conditions. In contrast, the =SONaCO_3_^2−^ complex was not detected, likely due to the low alkalinity of the system, which limits carbonate availability and competition for surface sites.

When comparing different POA dosages, a relatively higher surface site occupation was observed at 0.5 g/L compared to 2.0 g/L. This behavior may be attributed to the pH-buffering effect exerted by the higher number of available surface sites at increased dosages, which could shift the system away from the optimal pH range for phosphate retention.

#### 2.3.4. Effect of pH and Alkalinity on Phosphate Removal

**Figure 8:**
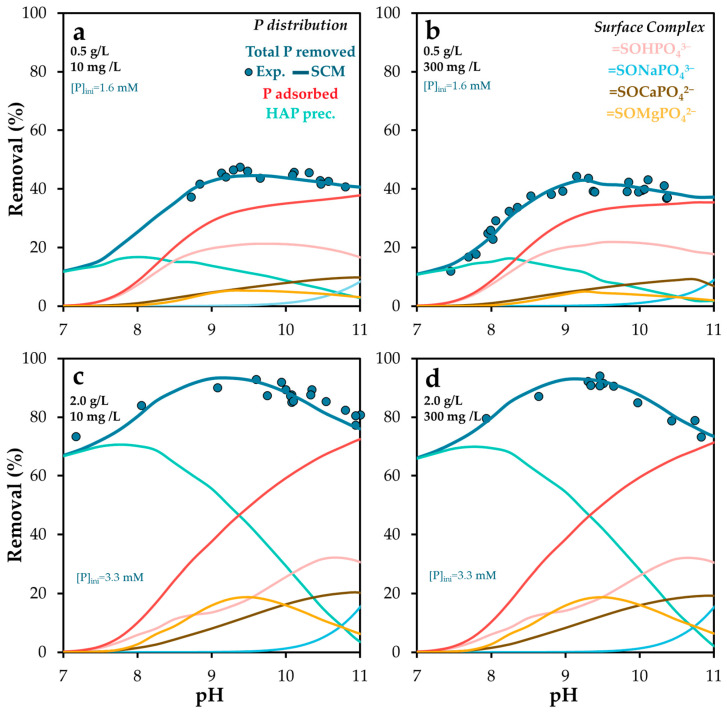
Experimental and Visual MINTEQ-simulated phosphorus removal and surface phosphorus speciation as a function of pH, at solid doses of (**a**,**b**) 0.5 and (**c**,**d**) 2.0 g/L, and alkalinity of (**a**,**c**) 10 mg/L and (**b**,**d**) 300 mg/L as CaCO_3_. The standard error of the mean ranged from 0.7 to 1.6%.

The influence of pH and alkalinity on phosphate removal was evaluated using two different POA dosages (0.5 and 2 g/L). Alkalinity was adjusted to 300 mg/L as CaCO_3_ by adding NaHCO_3_. Figure 8 and Figure S5 present the experimental and simulated results.

Phosphate adsorption exhibited clear pH dependence, indicating a predominant interaction with neutral surface sites, whose availability decreases under both acidic and highly alkaline conditions. This reduction is less pronounced at pH values above 9.5, likely due to the involvement of basic surface sites. These may facilitate phosphate binding through ternary complex formation [46], where cations released from POA act as bridging elements between the surface and phosphate species.

This behavior aligns with previous findings [62], which reported that phosphate adsorption on calcite is highly dependent on solution composition. Specifically, adsorption increases as carbonate activity decreases at constant pH.

Alkalinity plays a dual role in the system. On one hand, carbonate species exert a buffering effect that counteracts the pH-regulating capacity of the adsorbent. This buffering stabilizes the pH within optimal ranges for phosphate removal, suggesting that alkalinity control could be a useful strategy to optimize both removal efficiency and the dominant adsorption mechanism. Such control may also enhance the potential for post-treatment applications of the spent adsorbent by regulating its phosphorus release capacity.

A slight decrease in phosphate removal was observed at pH 10–11, which may be attributed to competitive adsorption between carbonate and phosphate anions. The competition between phosphate and other oxyanions has been widely reported [54,66]. For instance, Gustafson and Antelo [54] found that although arsenate and phosphate exhibit similar adsorption behavior on ferrihydrite in single-ion systems, arsenate is preferentially adsorbed in competitive systems, likely due to its stronger bidentate binding. Conversely, Xu et al. [66] reported that phosphate addition reduces arsenate adsorption on aluminum hydroxide surfaces, although it does not significantly alter arsenate surface speciation.

Mendez and Hiemstra [44] specifically investigated the competitive interaction between carbonate and phosphate on ferrihydrite. They demonstrated that pH-dependent phosphate adsorption as a function of carbonate concentration can successfully predict carbonate adsorption in single-ion systems. Moreover, they observed a higher affinity of carbonate for ferrihydrite at elevated pH and carbonate loadings, consistent with the trends observed in this study. However, the reductions in phosphate adsorption observed here were not substantial, suggesting that the competitive effect of carbonate is limited under the tested conditions.

**Figure 9:**
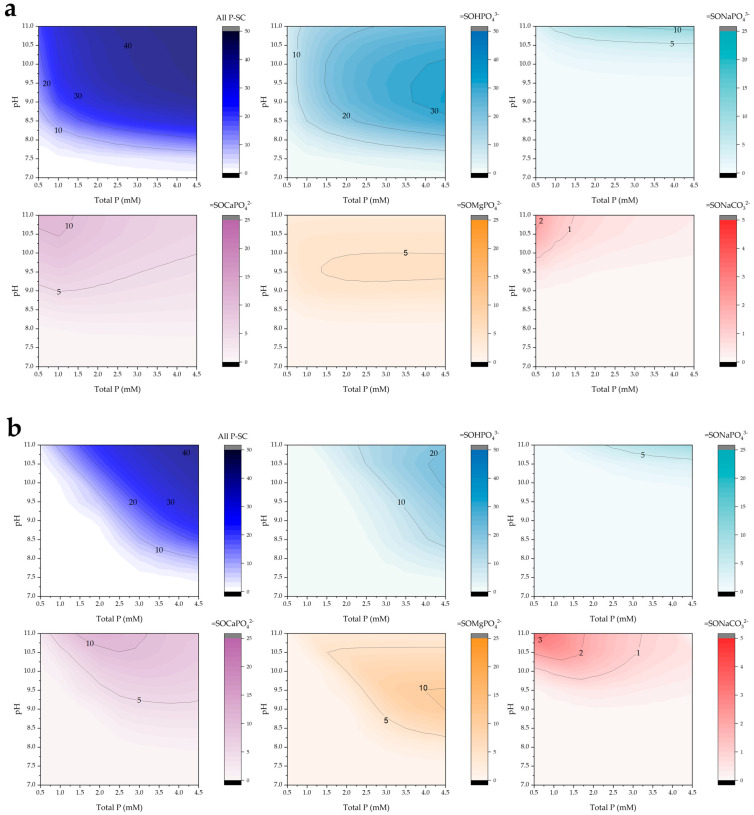
Simulated surface complex predominance (mg/g) as a function of pH and total phosphorus under alkalinity conditions (300 mg/L) for POA doses of (**a**) 0.5 and (**b**) 2.0 g/L.

Figure 9 illustrates the distribution of surface species for phosphate and carbonate considered in this study at POA doses of 0.5 g/L (Figure 9a) and 2.0 g/L (Figure 9b). The presence of alkalinity did not result in significant changes compared to conditions without added alkalinity (see Figure 7). This is primarily because the amount of carbonate retained as an adsorbed species (2–3 mg/g) was substantially lower than that of phosphate, and corresponded closely with the observed reduction in phosphate retention.

### 2.4. Model Validation

#### 2.4.1. Competitive and Synergetic Effects

To assess the predictive performance of the proposed model, additional experiments were carried out under extended chemical conditions. Using the previously calibrated parameters, simulation results were compared with experimental data.

**Figure 10:**
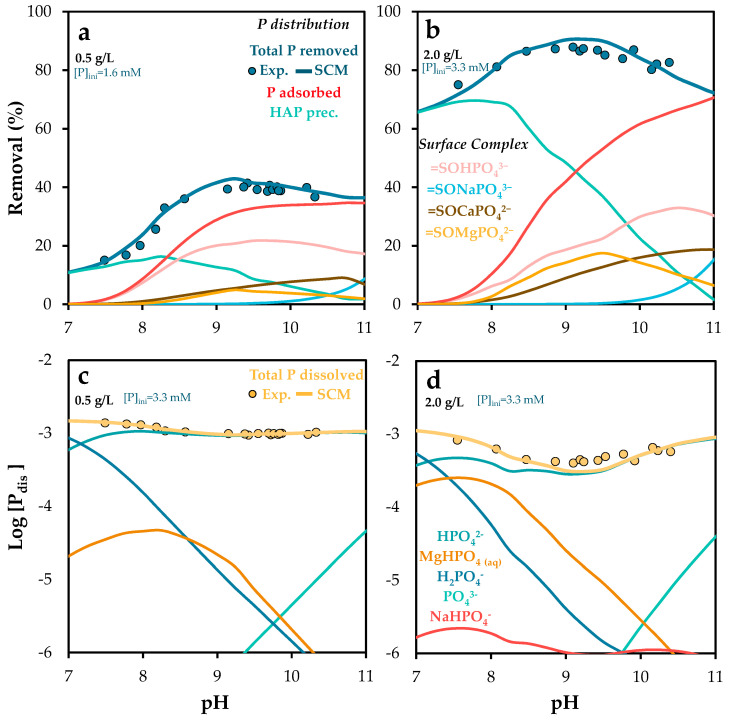
Experimental and Visual MINTEQ-simulated phosphorus removal, phosphorus dissolved, surface and aqueous phosphorus speciation as a function of pH, at solid doses of (**a**,**c**) 0.5 and (**b**,**d**) 2.0 g/L. Alkalinity adjusted at 450 mg/L as CaCO_3_. The standard error of the mean ranged from 0.6 to 1.7% (**a**,**b**) and from 0.01 to 0.04 (**c**,**d**).

Figure 10 shows the results obtained when alkalinity was increased to 450 mg/L. The model accurately predicted phosphate retention by POA across a wide range of pH and alkalinity levels, regardless of the adsorbent dose. As discussed earlier, the simulations confirmed that pH regulation is a key factor: surface complexation dominates at pH values above 8.5 (for 0.5 g/L) and 9.3 (for 2.0 g/L). Below these thresholds, precipitation mechanisms become more relevant, likely due to increased calcium release from the adsorbent (see Figure S6), which leads to supersaturation and the formation of calcium phosphate minerals such as hydroxyapatite [57,67]

The distribution of individual surface complexes is illustrated in Figure S7, where an increase in the formation of =SONaCO_3_^2−^ species is observed. This is attributed to the higher concentrations of sodium and carbonate in the system [44].

**Figure 11:**
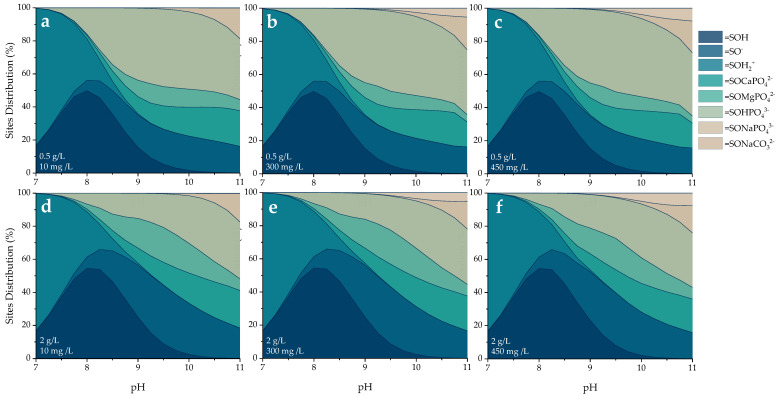
Visual MINTEQ-simulated sites and surface species distribution as a function of pH, at solid doses of (**a**–**c**) 0.5 and (**d**–**f**) 2.0 g/L, and alkalinity of (**a**,**d**) 10 mg/L, (**b**,**e**) 300, and (**c**,**f**) 450 mg/L as CaCO_3_.

Figure 11 presents the percentage distribution of active site occupation under different alkalinity conditions as a function of final pH.

The distribution of surface species at the active sites of the POA material, as a function of pH, reveals critical insights into the mechanisms governing phosphate retention. As shown in Figure 11, the speciation is strongly pH-dependent, reflecting the sequential protonation–deprotonation of surface hydroxyl groups and the formation of surface complexes with phosphate and carbonate ions.

At near-neutral pH values (7–8), the surface is primarily occupied by protonated species such as =SOH and =SOH_2_^+^, indicating a positively charged surface environment. This condition favors the electrostatic attraction of anionic species but limits the formation of phosphate surface complexes due to competition with protons. In the alkaline range (pH 9–11), surface complexation becomes the dominant mechanism. Species such as =SOHPO_4_^3−^ and =SOCaPO_4_^2−^, =SOMgPO_4_^2−^, in the presence of divalent cations, emerge as major contributors to surface speciation [46]. Under higher POA dosages (2 g/L), the formation of ternary complexes increases relative to =SOHPO_4_^3−^, possibly due to the greater availability of divalent cations released from the POA material. Additionally, the presence of =SONaCO_3_^2−^ suggests competitive adsorption with carbonate ions, which is consistent with the behavior of alkaline media where carbonate species are more abundant. This contribution becomes more significant as the alkalinity of the medium increases [44].

**Figure 12:**
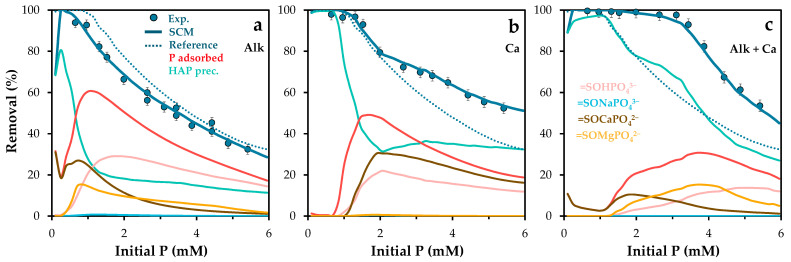
Experimental and Visual MINTEQ-simulated phosphorus removal and surface phosphorus speciation as a function of initial phosphorus concentration, under varying chemical conditions: (**a**) Addition of (**a**) NaHCO_3_ (alkalinity at 300 mg/L), (**b**) Ca(OH)_2_ (calcium at 120 mg/L), and (**c**) CaCO_3_ (300 mg/L). POA dose was adjusted at 1.0 g/L. Error bars represent the standard error of the mean.

To further validate the model, simulations were also performed under varying initial phosphate concentrations (Figure 12). As shown in Figure 12a, the model maintained strong predictive accuracy, particularly under competitive conditions at low phosphate concentrations [44].

The model was then tested under conditions of increased water hardness, by adding 120 mg/L of calcium to the phosphate solution. The presence of calcium was expected to enhance phosphate removal (see experimental isotherm in Figure S4b) through the formation of ternary surface complexes and calcium phosphate precipitates. Experimental results (Figure 12b) confirmed this synergistic effect, especially at higher phosphate concentrations. Simulations indicated a greater presence of the =SOCaPO_4_^2−^ complex and solid calcium phosphate phases. These findings highlight the potential of calcium as a functional additive in the development of new adsorbent materials [64].

The model was tested under combined alkalinity and hardness conditions (300 mg/L CaCO_3_). As shown in Figure 12c, phosphate retention increased significantly. This improvement is attributed to pH regulation that favors the formation of solid precipitates. According to Flower et al. [68], calcite can act as a major phosphorus sink through the formation of hydroxyapatite and amorphous calcium phosphate. At phosphate concentrations above 4 mM, the associated pH drop helps regulate retention mechanisms, resulting in removal efficiencies similar to those observed in Figure 12b. Simulations suggest that the increased availability of calcium promotes its incorporation into precipitated phases, reducing its participation in ternary complex formation.

**Figure 13:**
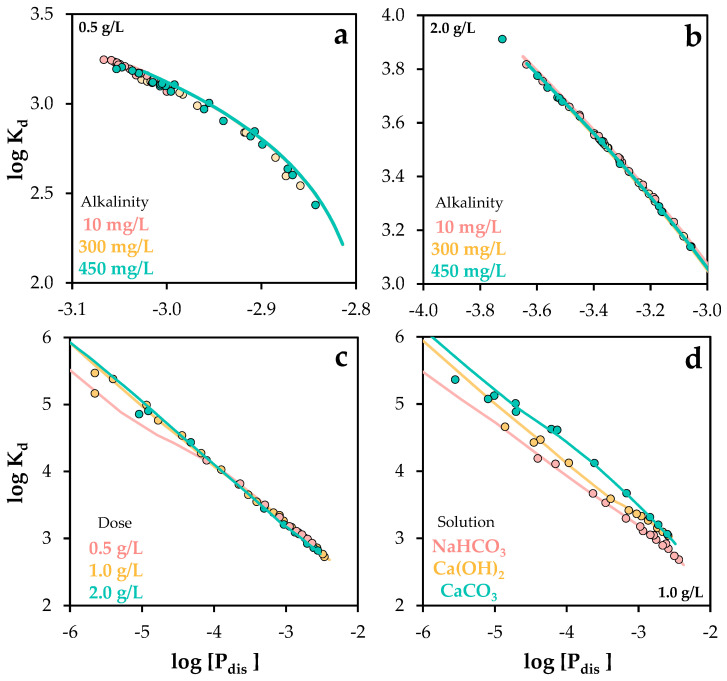
Experimental and Visual MINTEQ-simulated distribution coefficient values as a function of dissolved phosphorus concentration, under varying chemical conditions: (**a**,**b**) alkalinity changes, (**c**) phosphorus and POA dose changes, and (**d**) different solution conditions. The standard error of the mean ranged from 0.03 to 0.09 (**c**,**d**).

K_d_ values were employed in this study as a tool to assess the performance of the proposed model across a wide range of chemical conditions (see Figure 13).

In summary, the model demonstrated high predictive accuracy across a wide range of chemical conditions, including variations in adsorbent dose, phosphate concentration, pH, hardness, and alkalinity. This was quantitatively supported by the comparison of experimental and simulated distribution coefficients, with R^2^ values ranging from 0.92 to 0.99 across all scenarios.

#### 2.4.2. Sensitivity Analysis

Although accurate parameterization is essential in surface complexation modeling [69], few studies have systematically explored how sensitive model outputs are to variations in key parameters [70]. In this study, a sensitivity analysis was conducted to evaluate the influence of each parameter on model performance.

**Figure 14:**
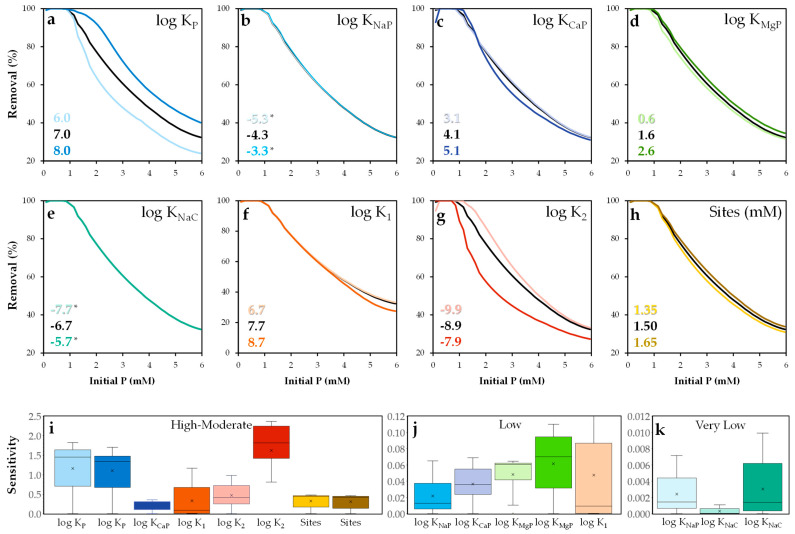
Sensitivity analysis of the NEM parameters (**a**–**h**) and relative sensitivity distribution values (**i**–**k**). * The colored lines are overlapped.

The analysis focused on the log K values of the proposed surface reactions (see Table 5) and the active site concentration. Default values from the Visual MINTEQ database were retained. Each log K value was individually varied by ±1 unit, and the active site concentration was adjusted by ±10%. The results are shown in Figure 14.

The model was most sensitive to the constants *K_P_* and K_2_ (Figure 14a,b,i), confirming the dominant role of the =SOHPO_4_^3−^ species in phosphate adsorption. The deprotonation of surface sites also had a significant impact on the availability of =SOH groups for phosphate binding. The K_1_ parameter exhibited some sensitivity at high phosphate concentrations, suggesting that surface protonation is not a dominant process under most conditions. In contrast, parameters such as K_CaP_ and the site concentration showed moderate sensitivity within the range of 0.2–0.5. The parameter K_MgP_ showed low effect, while the model showed minimal sensitivity to adsorption involving basic sites reactions, such as K_NaP_ and K_NaC_.

#### 2.4.3. Surface Analysis After Adsorption

**Figure 15:**
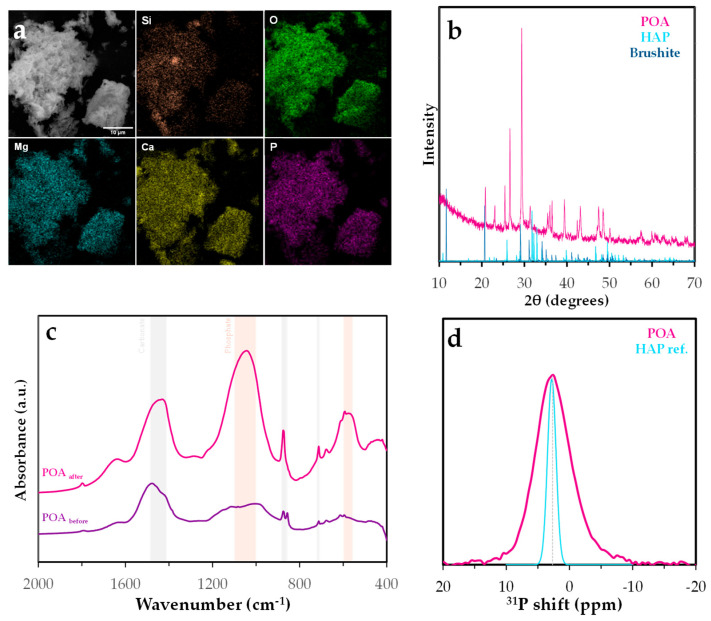
Characterization of phosphorus-saturated *Posidonia oceanica* ash: (**a**) FESEM analysis, (**b**) XRD diffractogram, (**c**) FTIR spectrum, and (**d**) ^31^P RMN spectrum.

To validate the proposed surface species, a combination of instrumental techniques was applied, including FESEM, FTIR, PXRD, and solid-state NMR. The results of these analyses are presented in Figure 15.

FTIR analysis revealed the simultaneous presence of characteristic phosphate phase bands (1100–1040 and 600–560 cm^−1^), indicative of PO_4_^3−^ groups [71]. Although other mineral phases such as quartz or calcite may produce overlapping signals, particularly in the 1040–1100 cm^−1^ region due to Si–O and P–O vibrations, the concurrent appearance of these phosphate bands strongly supports the presence of phosphate species rather than silicates. These bands are consistent with the formation of calcium phosphate phases such as hydroxyapatite (Ca_5_(PO_4_)_3_OH) or brushite (CaHPO_4_·2H_2_O) [72]. However, significant bands of carbonate mineral phases as calcite were also observed (1490–1410, 880–860, and 710 cm^−1^) [72].

Energy-dispersive X-ray spectroscopy (EDS) coupled with scanning electron microscopy (SEM) was used to assess the elemental distribution on the POA surface after phosphate adsorption. EDS mapping showed predominant signals for C, O, Si, Mg, Ca, and P, with minor contributions from Fe, Al, Na, and trace amounts of Cu and S. The ash matrix, rich in silicates and metal oxides, provides a heterogeneous surface favorable for multiple adsorption mechanisms. Phosphorus co-localized with Ca and Mg, suggesting the formation of insoluble precipitates or ternary surface complexes. The presence of Fe and Al oxides further supports phosphate retention via surface complexation with hydroxylated metal sites. The silica-rich matrix may also contribute to adsorption. The spatial distribution of phosphorus confirms effective retention through both precipitation and surface complexation.

X-ray diffraction (PXRD) analysis of POA after phosphate adsorption revealed diffraction peaks consistent with calcium phosphate phases, particularly hydroxyapatite and brushite. The repeated detection of these phases across multiple peaks suggests the formation of poorly crystalline or nanocrystalline precipitates on the adsorbent surface. The formation of hydroxyapatite-like phases indicates a chemisorption mechanism involving surface precipitation, a process widely reported in systems with available calcium ions from either the solution or the adsorbent itself [73]. These findings support the hypothesis that phosphate retention by POA involves not only surface complexation but also the nucleation of stable phosphate-containing mineral phases.

The ^31^P MAS NMR spectrum of the phosphorus-loaded POA (Figure 15d) exhibits a broad resonance centered at approximately 2.7 ppm, closely aligning with the sharp signal observed for the hydroxyapatite (HAP) reference [74]. This chemical shift is characteristic of orthophosphate groups in crystalline calcium phosphate phases. However, the broader linewidth (∼3.0 ppm) and reduced symmetry of the POA signal, compared to the narrower peak of the HAP reference (0.67 ppm), suggest a more disordered phosphorus environment, likely resulting from partial crystallinity or the coexistence of multiple phosphate species [74]. Such broadening may arise from structural imperfections, ionic substitutions (e.g., carbonate or metal ions), or interactions with the heterogeneous ash matrix. These results indicate that although HAP-like phases may be present, phosphorus is not exclusively incorporated as well-crystallized hydroxyapatite. Instead, amorphous phosphate phases or nanostructured calcium hydroxyapatite likely predominate [75]. This structural variability could influence phosphorus release behavior, which is critical for its application in nutrient recovery.

### 2.5. Phosphorus Release

Desorption experiments were conducted by replacing the residual solution, following adsorption equilibrium, with deionized water. As noted by So et al. [62], phosphate desorption is influenced by the concentration of dissolved phosphorus. To retain a minimal concentration of phosphate in solution, only 90% of the post-adsorption solution was replaced. This approach was applied to samples from previous model validation experiments, including the individual and combined presence of carbonate and calcium, as well as their absence.

Following desorption, a general decrease in pH was observed across all samples, ranging from 0.4 to 0.7 units. This trend is consistent with a reduction in the positive surface charge of POA, likely due to the formation of surface species that partially neutralize the charge. Consequently, P-loaded POA exhibits a diminished buffering capacity, which alters the final pH conditions and affects phosphorus speciation in the system, in agreement with previous findings [76].

**Figure 16:**
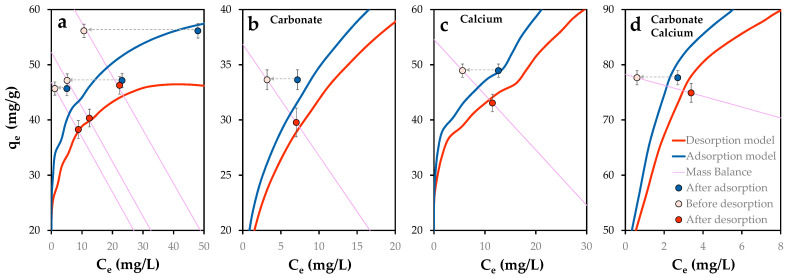
Phosphorus retention (mg/g) in function of dissolved phosphorus concentration from previously P-loaded POA (1 g/L) in variable chemical conditions: (**a**) Absence of co-ions, presence of single (**b**) carbonate and (**c**) calcium and (**d**) simultaneous presence of calcium and carbonate. Lines indicate modeled adsorption and desorption equilibrium curves and mass balance equation. Error bars represent the standard error of the mean. Arrows denote the change from previous equilibrium state to initial desorption condition.

To assess system evolution, the proposed model was used to simulate the new equilibrium scenario post-desorption. Figure 16 displays the experimental desorption behavior of phosphorus from POA, alongside the predicted adsorption and desorption equilibrium curves. Initial desorption conditions (white points) deviate from equilibrium, while final states (red points) align closely with the model predictions. The system’s trajectory follows the material balance equation (purple lines), confirming the model’s accuracy under varied chemical conditions.

Phosphorus release ranged from 8.0 mg (no co-ions), 5.9 (carbonate), 3.9 (calcium) to 2.7 mg (calcium and carbonate), corresponding to desorption ratios of 3.5–17.6%, consistent with previously reported ranges [77]. While Gérard [78] suggests that adsorption reduces phosphate leaching in acidic soils, this study observed lower leaching under alkaline conditions.

Although further investigation into desorption mechanisms is warranted, initial results support the potential reuse of exhausted POA as a source of phosphorus with extended release dynamics, offering a sustainable pathway for phosphorus recycling.

Overall, the model demonstrated strong predictive power, and the experimental progression also validates its applicability for describing phosphorus recovery from ash-based sorbents.

## 3. Materials and Methods

### 3.1. Adsorbent Preparation

Raw *Posidonia oceanica* litter was collected from beaches along the northern coast of Alicante, Spain. The fibrous and ball-like components were cut into 5–10 mm fragments and repeatedly washed with deionized water to remove residual salts and sand. The cleaned material was then dried at 60 °C for 24 h and subsequently incinerated at 550 °C for 2 h in a muffle furnace. The resulting ash was washed again with deionized water at a solid-to-liquid ratio of 50 g/L until constant conductivity was achieved. The solid was then separated and dried at 105 °C for 12 h. The dried *Posidonia oceanica* ash was designated as POA and stored in airtight containers for further use or modification.

### 3.2. Characterization and Analytical Methods

#### 3.2.1. Adsorbent Characterization

To characterize the adsorbent, various analytical techniques were employed. Major and trace elements in the solid were determined after dissolution in an aqueous mixture of HNO_3_/HCl/HF by Inductively Coupled Plasma (ICP) analysis using a Varian 715-ES ICP optical emission spectrometer (Varian Inc., Palo Alto, CA, USA). The elemental composition of POA was further analyzed using a CHNS-O Elemental Analyzer (EA-1108, Fisons, Loughborough, UK).

Crystalline phases present in the biochar before and after phosphate adsorption were identified via X-ray diffraction (XRD) using a Philips X’Pert multisample diffractometer equipped with a graphite monochromator (Philips, Amsterdam, The Netherlands), operating at 40 kV and 35 mA with Cu Kα radiation (λ = 0.1542 nm).

Textural properties of POA were assessed through N_2_ adsorption–desorption isotherms measured at 77 K using a Micromeritics ASAP 2020 system (Micromeritics, Norcross, GA, USA).

Morphological analysis was conducted using field-emission scanning electron microscopy (FESEM) with a ZEISS Ultra 55 microscope (ZEISS, Oberkochen, Germany), operated at 10 kV and 20 µA. Images were acquired in combined secondary and backscattered electron modes at a working distance of 10–15 mm. Elemental composition and distribution were further examined before and after phosphate adsorption using energy-dispersive X-ray spectroscopy (EDS) mapping.

Fourier-transform infrared spectroscopy (FTIR) analyses were performed using a Bruker Alpha II spectrometer over the range of 500–4000 cm^−1^ (Bruker, Billerica, MA, USA).

To characterize the solid sample after adsorption, the 31P MAS NMR spectrum was recorded by using a 3.2 mm probe spinning at 20 kHz using a π/2 pulse length of 3.7 μs with SPINAL proton decoupling and a recycle delay of 20 s.

#### 3.2.2. Surface Charge and Point of Zero Charge

Surface charge was determined using the titration method [79]. Potentiometric titrations of POA were carried out at a controlled temperature of 293 K in a sealed glass bottle equipped with ports for a temperature probe, microburette, and pH/conductivity electrodes. A suspension of 75 mg of POA was equilibrated for 1 h under continuous stirring at a dosage of 1 g/L and an ionic strength of 0.002 N NaCl. To assess the effects of adsorbent concentration and ionic strength, additional experiments were conducted at 0.5 g/L and 0.02 N NaCl, respectively. Furthermore, POA samples previously exposed to phosphate solutions (100 and 200 mg/L for 96 h) were also titrated. Once the pH stabilized, titration was performed by incremental addition of 0.05 mL of 0.1 N HCl.

Surface charge density (σ_0_, in C/m^2^) was calculated using Equation (1):

(1) $$\sigma_{0}=\frac{F\cdot \left(\left[C_{a}\right]-\left[C_{b}\right]+\left[H^{+}\right]-\left[{OH}^{-}\right]\right)}{m\cdot S}$$

where F is the Faraday constant (C/mol), C_a_ and C_b_ are the concentrations (mol/L) of acid and base added, [H^+^] and [OH^−^] are derived from the measured pH, and m and S are the mass (g) and specific surface area (m^2^/g) of the solid.

The point of zero charge (pH_PZC_) was determined using the solid addition method [80]. A 0.01 N NaCl solution was adjusted to initial pH values ranging from 2 to 11 using 0.1 N HCl or NaOH. Then, 50 mg of POA was added to 50 mL of the adjusted solution in a 100 mL stoppered Erlenmeyer flask and stirred for 24 h at 293 K. The pH_PZC_ was obtained from the plot of ΔpH (pH_final_ − pH_initial_) vs. pH_initial_.

#### 3.2.3. Water Analysis

Residual phosphate concentrations in solution after adsorption and desorption experiments were measured using the vanadomolybdophosphoric acid colorimetric method [81] with a UV–Vis spectrophotometer (UV-1600PC, VWR, Radnor, PA, USA) at 410 nm. The concentrations of the main ions present in the aqueous matrices were below the interference thresholds specified in the method. To validate the accuracy of the method, the results were compared with those obtained via ICP analysis. The differences between the two methods were consistently less than 0.1 mg/L and 0.5%.

Calcium and magnesium concentrations in the solution were determined by atomic absorption spectroscopy (AAS) using a Varian SpectrAA-220 instrument (Varian Inc., Palo Alto, CA, USA).

Elements leached from POA during adsorption and desorption were quantified by ICP analysis of 0.45 µm-filtered solutions using a Varian 715-ES ICP optical emission spectrometer.

### 3.3. Batch Experiments

#### 3.3.1. Phosphate Removal

Batch experiments were conducted to evaluate the total phosphate removal capacity of POA. A stock phosphorus solution (200 mg/L) was prepared by dissolving analytical-grade sodium phosphate dibasic (NaH_2_PO_4_) in deionized water. To assess the effect of phosphate concentration, dilutions ranging from 5 to 200 mg/L were prepared.

Adsorption tests were performed using 50 mL of phosphate solution and varying the solid-to-liquid ratio (0.5, 1, and 2 g/L) in 100 mL stoppered Erlenmeyer flasks. Suspensions were agitated at 180 rpm and maintained at a controlled temperature of 293 K for 96 h. To determine the appropriate contact time required to reach equilibrium, preliminary experiments were conducted in which the contact duration was varied within the range of 24 to 120 h. After contact, POA and solution were separated by centrifugation (5000 rpm, 5 min) followed by filtration through a 0.45 µm membrane syringe filter. Conductivity and pH were measured using Crison instruments, and residual phosphate concentrations were analyzed. All experiments were conducted in triplicate.

In experiments under variable chemical conditions, ionic strength, alkalinity, and water hardness were adjusted by adding analytical-grade salts: NaCl, NaHCO_3_, Ca(OH)_2_, MgCl_2_, and CaCO_3_. For pH-edge experiments, the initial pH was adjusted using 0.1 N HCl or NaOH solutions.

Phosphate removal capacity (*q_e_*, mg P/g), removal efficiency (*R_e_*), and distribution coefficient (*K_d_*) were calculated using Equations (2)–(4):

(2) $$q_{e}=\frac{\left(C_{0}-C_{e}\right)\cdot V}{m}$$

(3) $$R_{e}=\frac{\left(C_{0}-C_{e}\right)}{C_{0}}\cdot 100$$

(4) $$K_{d}=\frac{q_{e}}{C_{e}}$$

where *C*_0_ and *C_e_* are the initial and equilibrium phosphate concentrations (mg/L), *V* is the solution volume (L), and *m* is the mass of adsorbent (g).

Thermodynamic adsorption models are particularly well suited for deriving distribution coefficients (*K_d_*) as a function of environmental variables, as they allow the coupling of adsorption behavior with changing chemical conditions.

#### 3.3.2. Phosphate Release

After experiments under variable chemical conditions, including the presence of ionic strength, alkalinity, calcium and CaCO_3_, around 80–90% of solution was replaced by deionized water to reduce phosphate concentration in solution. Suspensions were agitated at 180 rpm and maintained at 293 K for 24 h. Samples were filtered as described for adsorption tests, and phosphate and major cations leached into the solution were quantified.

### 3.4. Surface Complexation Modeling

To determine the chemical species present at equilibrium, thermodynamic calculations were performed using the Visual MINTEQ 3.1 software. To represent the adsorption mechanism at the solid–liquid interface, the non-electrostatic model (NEM) was employed, as it is the simplest model and requires fewer parameters. NEM simulates surface complexation reactions similar to solution complexation, without accounting for electrostatic interactions. Surface hydroxyl groups were considered as active sites, denoted as =SOH, and described with a 2-pK model that can undergo protonation or deprotonation according to the following reactions (5) and (6):

(5) $$=\mathrm{SOH}\;+\;H^{+}\;↔\;=\;{{\mathrm{SOH}}_{2}}^{+}\;\;K_{1}=\frac{\left[{=SOH}_{2}^{+}\right]}{\left[=SOH\right]\left\{H^{+}\right\}}$$

(6) $$=\mathrm{SOH}\;↔\;=\;{\mathrm{SO}}^{-}+H^{+}\;\;K_{2}=\frac{\left[{=SO}^{-}\right]\cdot \left\{H^{+}\right\}}{\left[=SOH\right]}$$

Phosphate adsorption by POA was simulated using NEM integrated in Visual MINTEQ. The concentration of active sites was determined via acid–base titration, as previously described. The equilibrium constant for surface complexation was defined according to stoichiometry, exemplified by reaction (7).

(7) $$=\mathrm{SOH}+{\mathrm{PO}}_{4}^{3-}\;↔\;=\;{\mathrm{SOHPO}}_{4}^{3-}\;\;K_{P}=\frac{\left[{=SOHPO}_{4}^{3-}\right]}{\left[=SOH\right]\left\{{=PO}_{4}^{3-}\right\}}$$

To assess the potential formation of solid precipitates, the saturation index (*SI*) of possible phases was calculated using Equation (8)

(8) $$SI=Log\left(\frac{IAP}{K_{PS}}\right)$$

where *IAP* is the ion activity product and *K_ps_* is the solubility product (see Table S3).

The changes in pH and ion compositions by using different dosages of POA were predicted by using the Multi-problem/Sweep function.

### 3.5. Model Validation and Sensitivity Analysis

To validate the model, additional adsorption experiments were conducted under modified chemical conditions: increased alkalinity (300 mg/L, adjusted with NaHCO_3_), increased hardness (calcium at 120 mg/L, adjusted with Ca(OH)_2_), and combined effects of calcium and carbonate ions (300 mg/L CaCO_3_), following the same experimental procedure as described above. Model performance was evaluated by comparing experimental and simulated results using the coefficient of determination (R^2^) and root mean square error (RMSE).

To assess model sensitivity to adsorption constants and active site concentration, the log K values were varied by ±1 unit and the active site concentration by ±10%. The relative sensitivity (*S_k_*) of each parameter k was calculated using Equation (9):

(9) $$S_{k}=\frac{\left(C-C_{R}\right)/C_{R}}{\left(k-k_{R}\right)/k_{R}}$$

where *C* and *k* are the modified values of the calculated variable and the parameter, respectively, and *C_R_* and *k_R_* are their reference values.

## 4. Conclusions

A novel adsorbent material derived from *Posidonia oceanica* residues was successfully developed and characterized, demonstrating high efficiency for phosphate removal from aqueous solutions. The material exhibited a total phosphate removal capacity ranging from 35 to 58 mg/g, surpassing many other materials reported in the literature. Its adsorption performance was found to be strongly influenced by the chemical conditions of the medium: it increased with phosphate concentration and water hardness, decreased with alkalinity and adsorbent dosage, and reached optimal performance at pH values between 9.0 and 9.5. A thermodynamic surface complexation modeling approach, based on a NEM proposal, was developed and supported by instrumental solid-phase analyses to elucidate the dominant phosphate retention mechanisms. The resulting model showed strong agreement with experimental data and could be readily integrated into broader thermodynamic frameworks. The model was validated against experimental results, and its predictive reliability was assessed, confirming its robustness under varying chemical conditions. Furthermore, preliminary evaluation of the spent POA material suggests its potential for nutrient recovery applications, particularly through prolonged phosphorus release, contributing to circular economy strategies in water treatment.

## Figures and Tables

**Table 1:** Physicochemical characterization of *Posidonia oceanica* ash.

Surface Properties				
	BET Area	External Area	Micropore	pH_PZC_ ^a^
	(m^2^/g)	(%)	(cm^3^/g)	
	13.25	86	0.00084	9.1
**Elementary Analysis (wt. %)**				
	Carbon	Nitrogen	Hydrogen	Sulfur
	3.42	0.01	0.48	1.85
**Chemical Composition (wt. %)**				
	P_2_O_5_	0.39	MgO	8.44
	SiO_2_	23.64	K_2_O	0.89
	Al_2_O_3_	2.67	Na_2_O	1.62
	Fe_2_O_3_	5.31	CaO	14.48

^a^ The pH_PZC_ was determined using the solid addition method at a solid concentration of 0.01 wt. %.

**Table 2:** Comparison of phosphate removal capacity of various bio-based adsorbents. The literature values are indicative, due to the variability in experimental setups.

Precursor Material	Adsorbent	Dose (g/L)	q (mg/g)	Reference
*Posidonia oceanica* fiber	Raw	10	3.0 ^a^	[27]
K-clay, corn starch, and calcium oxide	Raw	4	4.4 ^a^	[28]
*Posidonia oceanica* fiber	Raw	2	5.0 ^a^	[29]
Sesame straw	Biochar	2	9.4 ^b^	[30]
Peanut shell	Biochar	1	7.6 ^b^	[31]
Municipal waste	Biochar	1	13.1 ^a^	[32]
Maize straw	Biochar	100	9.5 ^b^	[33]
Sewage sludge	Biochar	1	15.2 ^a^	[34]
Cocoa pod husk	Biochar	5	5.8 ^a^	[35]
Holm oak wood	Biochar	1	4.1 ^b^	[36]
Walnut shell	Biochar	8	3.2 ^a^	[37]
Walnut shell	Biochar	2	6.4 ^b^	[38]
Prosopis juliflora	AC	4	6.7 ^b^	[39]
Lignin	Magnetic AC	6.7	21.2 ^b^	[40]
Rice straw	Ash	10	3.2 ^a^	[41]
Reed	Ash	2.5	14.7 ^a^	[41]
Amine-functionalized rice husk	Ash	0.6	13.4 ^b^	[42]
*Posidonia oceanica* fiber	Ash	0.5–2	33.5–58.7 ^a^	This study

^a^ Precipitation-inclusive phosphate removal capacity. ^b^ Purely adsorptive system.

**Table 3:** Components and other species used in the model and reaction stoichiometry.

		Components								
Species	log K	CO_3_^2−^	Ca^2+^	H^+^	H_2_O	K^+^	Mg^2+^	Na^+^	PO_4_^3−^	Si ^1^
CaCO_3 (aq)_	3.22	1	1	0	0	0	0	0	0	0
CaH_2_PO_4_^+^	20.92	0	1	2	0	0	0	0	1	0
CaHCO_3_^+^	11.43	1	1	1	0	0	0	0	0	0
CaHPO_4 (aq)_	15.04	0	1	1	0	0	0	0	1	0
CaOH^+^	−12.70	0	1	−1	1	0	0	0	0	0
CaPO_4_^−^	6.46	0	1	0	0	0	0	0	1	0
H_2_CO_3 (aq)_	16.68	1	0	2	0	0	0	0	0	0
H_2_PO_4_^−^	19.57	0	0	2	0	0	0	0	1	0
H_2_SiO_4_^2−^	−23.04	0	0	−2	0	0	0	0	0	1
H_3_PO_4_	21.72	0	0	3	0	0	0	0	1	0
H_3_SiO_4_^−^	−9.84	0	0	−1	0	0	0	0	0	1
HCO_3_^−^	10.33	1	0	1	0	0	0	0	0	0
HPO_4_^2−^	12.38	0	0	1	0	0	0	0	1	0
K_2_HPO_4 (aq)_	13.50	0	0	1	0	2	0	0	1	0
K_2_PO_4_^−^	2.26	0	0	0	0	2	0	0	1	0
KH_2_PO_4 (aq)_	19.87	0	0	2	0	1	0	0	1	0
KHPO_4_^−^	13.26	0	0	1	0	1	0	0	1	0
KOH _(aq)_	−13.76	0	0	−1	1	1	0	0	0	0
KPO_4_^2−^	1.43	0	0	0	0	1	0	0	1	0
Mg_2_CO_3_^2+^	3.59	1	0	0	0	0	2	0	0	0
MgCO_3 (aq)_	2.92	1	0	0	0	0	1	0	0	0
MgHCO_3_^+^	11.34	1	0	1	0	0	1	0	0	0
MgHPO_4 (aq)_	15.18	0	0	1	0	0	1	0	1	0
MgOH^+^	−11.42	0	0	−1	1	0	1	0	0	0
MgPO_4_^−^	4.65	0	0	0	0	0	1	0	1	0
Na_2_HPO_4 (aq)_	13.32	0	0	1	0	0	0	2	1	0
Na_2_PO_4_^−^	2.59	0	0	0	0	0	0	2	1	0
NaCO_3_^−^	1.27	1	0	0	0	0	0	1	0	0
NaH_2_PO_4 (aq)_	19.87	0	0	2	0	0	0	1	1	0
NaHCO_3 (aq)_	10.03	1	0	1	0	0	0	1	0	0
NaHPO_4_^−^	13.45	0	0	1	0	0	0	1	1	0
NaOH _(aq)_	−13.90	0	0	−1	1	0	0	1	0	0
NaPO_4_^2−^	1.43	0	0	0	0	0	0	1	1	0
OH^−^	−14.00	0	0	−1	1	0	0	0	0	0
CO_2 (g)_	18.16	1	0	2	−1	0	0	0	0	0

^1^ Si refers to H_4_SiO_4_.

**Table 4:** Surface complexation species considered in the model and reaction stoichiometry.

Surface Species	K_a_	log K	=SOH	H^+^	Ca^2+^	Mg^2+^	Na^+^	PO_4_^3−^	CO_3_^2−^
Surface (de)protonation									
=SOH_2_^+^	K_1_	7.7	1	1	0	0	0	0	0
=SO^−^	K_2_	−8.9	1	−1	0	0	0	0	0
Surface complexation									
=SOHPO_4_^3−^	K_P_	7.0	1	0	0	0	0	1	0
=SONaPO_4_^3−^	K_NaP_	−4.3	1	−1	0	0	1	1	0
=SOCaPO_4_^2−^	K_CaP_	4.1	1	−1	1	0	0	1	0
=SOMgPO_4_^2−^	K_MgP_	1.6	1	−1	0	1	0	1	0
=SONaCO_3_^2−^	K_NaC_	−6.7	1	−1	0	0	1	0	1

**Table 5:** Experimental values in titrations and adsorbent (de)protonation parameters calculated.

Parameter		POA Before Adsorption			POA After Adsorption	
		1	2	3	4	5
General						
	Solid Concentration (g/L)	0.5	1.0	1.0	1.0	1.0
	Ionic Strength (N)	0.002	0.003	0.019	0.002	0.002
	Initial P-PO_4_ (mg/L)	--	--	--	100	200
NEM Parameters						
	log K_1_	7.6	8.0	7.9	7.4	7.2
	log K_2_	−9.8	−9.5	−9.3	−8.1	−7.8
	pH_PZC (calculated)_	8.75	8.75	8.62	7.77	7.50
	Sites Concentration (mM)	0.9	1.7	2.8	1.2	1.1
	Sites Density (mol/g)	0.0018	0.0017	0.0028	0.0012	0.0011
Error Functions						
	RMSE	0.0213	0.0317	0.0609	0.0152	0.0472
	R^2^ Coefficient	0.9969	0.9989	0.9961	0.9901	0.9812

## Data Availability

Data is contained within the article or Supplementary Material.

## Supplementary Materials

The following supporting information can be downloaded at: https://www.mdpi.com/article/10.3390/molecules30173639/s1. Table S1. Experimental conditions considered in phosphate adsorption batch experiments. Table S2. Components in water solution after POA contact at 10 g/L after 5 days. Concentration (in mg/L) were determined by ICP chemical analysis. Table S3. Possible solid phases considered in the model and reaction stoichiometry. Figure S1. Experimental and Protofit-simulated titration curves of Posidonia Ash under different chemical conditions: (a–c) before adsorption, (d–e) after adsorption and (f) background electrolyte. Figure S2. Zero Charge Point as a function of solid dose for different determination methods. Error bars represent the standard error of the mean. Figure S3. Effect of contact time on phosphate adsorption capacity for different chemical conditions: presence of phosphate (1), high adsorbate/solid ratio (2), alkalinity (3) and water hardness (4). Alkalinity and water hardness expressed as CaCO3. Error bars represent the standard error of the mean. Figure S4. Experimental phosphorus removal capacity at equilibrium as a function of equilibrium dissolved phosphorus concentration, under varying chemical conditions: (a) phosphorus and POA dose changes and (b) different solution conditions. The standard error of the mean ranged from 0.008 to 0.053. Figure S5. Experimental and Visual MINTEQ-simulated phosphorus dissolved and aqueous phosphorus speciation as a function of pH, at solid doses of (a,b) 0.5 and (c,d) 2.0 g/L, and alkalinity of (a,c) 10 mg/L and (b,d) 300 mg/L as CaCO3. The standard error of the mean ranged from 0.012 to 0.043. Figure S6. Calcium released by POA adsorbent at different doses as a function of (a) initial and (b) final system pH. The standard error of the mean ranged from 0.016 to 0.049. Figure S7. Simulated surface complex predominance (mg/g) as a function of pH and total phosphorus under alkalinity conditions (450 mg/L) for POA doses of (a) 0.5 and (b) 2.0 g/L.

## Abbreviations

The following abbreviations are used in this manuscript:

AAS	Atomic Absorption Spectroscopy
EDS	Energy-Dispersive X-Ray Spectroscopy
FTIR	Fourier-Transform Infrared Spectroscopy
HAP	Hydroxyapatite
ICP	Inductively Coupled Plasma
NEM	Non-Electrostatic Model
NMR	Nuclear Magnetic Resonance
POA	*Posidonia oceanica* Ash
PZC	Point of Zero Charge
SCM	Surface Complexation Model
SEM	Scanning Electron Microscopy
XRD	X-Ray Diffraction
