# Supplementary material for: Sustainable Valorization of Posidonia Waste Ash for Phosphate Removal: A Surface Complexation Approach Under Variable Water Chemistry
Source: Molecules. 2025 Sep 6;30(17):3639. doi: 10.3390/molecules30173639 (PMC12430204; doi:10.3390/molecules30173639)
Supplement: Supplementary file 1 [file molecules-30-03639-s001.zip › molecules-3823402-supplementary.pdf]

Supplementary Material

Sustainable Valorization of *Posidonia* Waste Ash for Phosphate Removal: A Surface Complexation Approach under Variable Water Chemistry

Jesús Mengual [1,*] and Juan A. González [2]

[1] Instituto de Tecnología Química, Universitat Politècnica de València – Consejo Superior de Investigaciones Científicas, Avenida de los Naranjos s/n, València, 46022, Spain; jemencu@itq.upv.es
[2] Institut Universitari d'Investigació d'Enginyeria de l'Aigua i Medi Ambient, IIAMA, Universitat Politècnica de València, Camí de Vera s/n, 46022, Valencia, Spain; juagonr1@hma.upv.es
* Correspondence: jemencu@itq.upv.es

Corresponding Author:

Jesús Mengual, PhD.

Email: jemencu@itq.upv.es

This *Supporting Information* includes

Number of Tables: 3

Number of Figures: 7

List of Tables

Table S 1. Experimental conditions considered in phosphate adsorption batch experiments. 3

Table S 2. Components in water solution after POA contact at 10 g/L after 5 days. Concentration (in mg/L) were determined by ICP chemical analysis. 4

Table S 3. Possible solid phases considered in the model and reaction stoichiometry. .. 5

List of Figures

Figure S 1. Experimental and Protofit-simulated titration curves of *Posidonia* Ash under different chemical conditions: (a –c) before adsorption, (d – e) after adsorption and (f) background electrolyte. 6

Figure S 2. Zero Charge Point as a function of solid dose for different determination methods. Error bars represent the standard error of the mean. 7

Figure S 3. Effect of contact time on phosphate adsorption capacity for different chemical conditions: presence of phosphate (1), high adsorbate/solid ratio (2), alkalinity (3) and water hardness (4). Alkalinity and water hardness expressed as $CaCO_3$. Error bars represent the standard error of the mean. 8

Figure S 4. Experimental phosphorus removal capacity at equilibrium as a function of equilibrium dissolved phosphorus concentration, under varying chemical conditions: (a) phosphorus and POA dose changes and (b) different solution conditions. The standard error of the mean ranged from 0.008 to 0.053. 9

Figure S 5. Experimental and Visual MINTEQ-simulated phosphorus dissolved and aqueous phosphorus speciation as a function of pH, at solid doses of (a, b) 0.5 and (c, d) 2.0 g/L, and alkalinity of (a, c) 10 mg/L and (b, d) 300 mg/L as $CaCO_3$. The standard error of the mean ranged from 0.012 to 0.043. 10

Figure S 6. Calcium released by POA adsorbent at different doses as a function of (a) initial and (b) final system pH. The standard error of the mean ranged from 0.016 to 0.049. 11

Figure S 7. Simulated surface complex predominance (mg/g) as a function of pH and total phosphorus under alkalinity conditions (450 mg/L) for POA doses of (a) 0.5 and (b) 2.0 g/L. 12

Table S1. Experimental conditions considered in phosphate adsorption batch experiments.

Variable Study	Experimental Conditions					
	**Phosphorus** (mg $P\text{-}PO_4$/L)	**Solid** (g/L)	**pH**	**Alkalinity**[a] (mg/L)	**Hardness**[a] (mg/L)	**Temperature** (ºC)
Effect of …						
Initial $P\text{-}PO_4$	50 to 150	1.0	8.7-9.3	0	0	20
Solid Concentration	100	0.5 to 2.0	7.8-9.8	0	0	20
pH	100	2.0	7.2 to 11.1	0	0	20
Alkalinity	50	0.5	9.9-10.3	0 to 450	0	20
Water Hardness	50	1.0	8.0-8.3	0	0 to 600	20

[a] Alkalinity and Hardness measured as $CaCO_3$

Table S2. Components in water solution after POA contact at 10 g/L after 5 days. Concentration (in mg/L) were determined by ICP chemical analysis.

pH	P	Si	Fe	Mg	Ca	Al	Na	K
7.1	0	81	0	323	1254	0	58	9
8.4	0	26	0	147	303	0	27	10
10.8	0	17	0	21	422	0	32	4

Table S3. Possible solid phases considered in the model and reaction stoichiometry.

Solid	log K	Components								
		CO_3^{2-}	Ca^{2+}	H^+	H_2O	K^+	Mg^{2+}	Na^+	PO_4^{3-}	Si[1]
Aragonite	8.31	1	1	0	0	0	0	0	0	0
Artinite	-9.96	1	0	-2	5	0	2	0	0	0
Brucite	-17.44	0	0	-2	2	0	1	0	0	0
$Ca_3(PO_4)_2$ (am1)	25.22	0	3	0	0	0	0	0	2	0
$Ca_3(PO_4)_2$ (am1)	27.99	0	3	0	0	0	0	0	2	0
$Ca_3(PO_4)_2$ (beta)	29.08	0	3	0	0	0	0	0	2	0
$Ca_4H(PO_4)_3{\cdot}3H_2O$ (s)	47.64	0	4	1	3	0	0	0	3	0
$CaCO_3{\cdot}xH_2O$ (s)	7.11	1	1	0	1	0	0	0	0	0
$CaHPO_4$ (s)	19.37	0	1	1	0	0	0	0	1	0
$CaHPO_4{\cdot}2H_2O$ (s)	19.06	0	1	1	2	0	0	0	1	0
Calcite	8.45	1	1	0	0	0	0	0	0	0
Chalcedony	3.61	0	0	0	-2	0	0	0	0	1
Chrysotile	-32.79	0	0	-6	1	0	3	0	0	2
Cristobalite	3.41	0	0	0	-2	0	0	0	0	1
Dolomite (disordered)	16.40	2	1	0	0	0	1	0	0	0
Dolomite (ordered)	16.97	2	1	0	0	0	1	0	0	0
Huntite	29.65	4	1	0	0	0	3	0	0	0
Hydromagnesite	8.11	4	0	-2	6	0	5	0	0	0
Hydroxyapatite	44.33	0	5	-1	1	0	0	0	3	0
Lime	-33.28	0	1	-2	1	0	0	0	0	0
Magnesite	7.52	1	0	0	0	0	1	0	0	0
$Mg(OH)_2$ (active)	-18.79	0	0	-2	2	0	1	0	0	0
$Mg_3(PO_4)_2$ (s)	23.28	0	0	0	0	0	3	0	2	0
$MgCO_3{\cdot}5H_2O$ (s)	4.54	1	0	0	5	0	1	0	0	0
MgHPO4·3H2O (s)	18.17	0	0	1	3	0	1	0	1	0
Natron	1.51	1	0	0	10	0	0	2	0	0
Nesquehonite	4.60	1	0	0	3	0	1	0	0	0
Periclase	-22.04	0	0	-2	1	0	1	0	0	0
Portlandite	-23.09	0	1	-2	2	0	0	0	0	0
Quartz	4.07	0	0	0	-2	0	0	0	0	1
Sepiolite	-16.10	0	0	-4	-0.5	0	2	0	0	3
Sepiolite (A)	-18.78	0	0	-4	-0.5	0	2	0	0	3
SiO_2 (am,gel)	2.75	0	0	0	-2	0	0	0	0	1
SiO_2 (am,ppt)	2.79	0	0	0	-2	0	0	0	0	1
Thermonatrite	-0.67	1	0	0	1	0	0	2	0	0
Vaterite	7.87	1	1	0	0	0	0	0	0	0

[1] Si refers to H_4SiO_4.

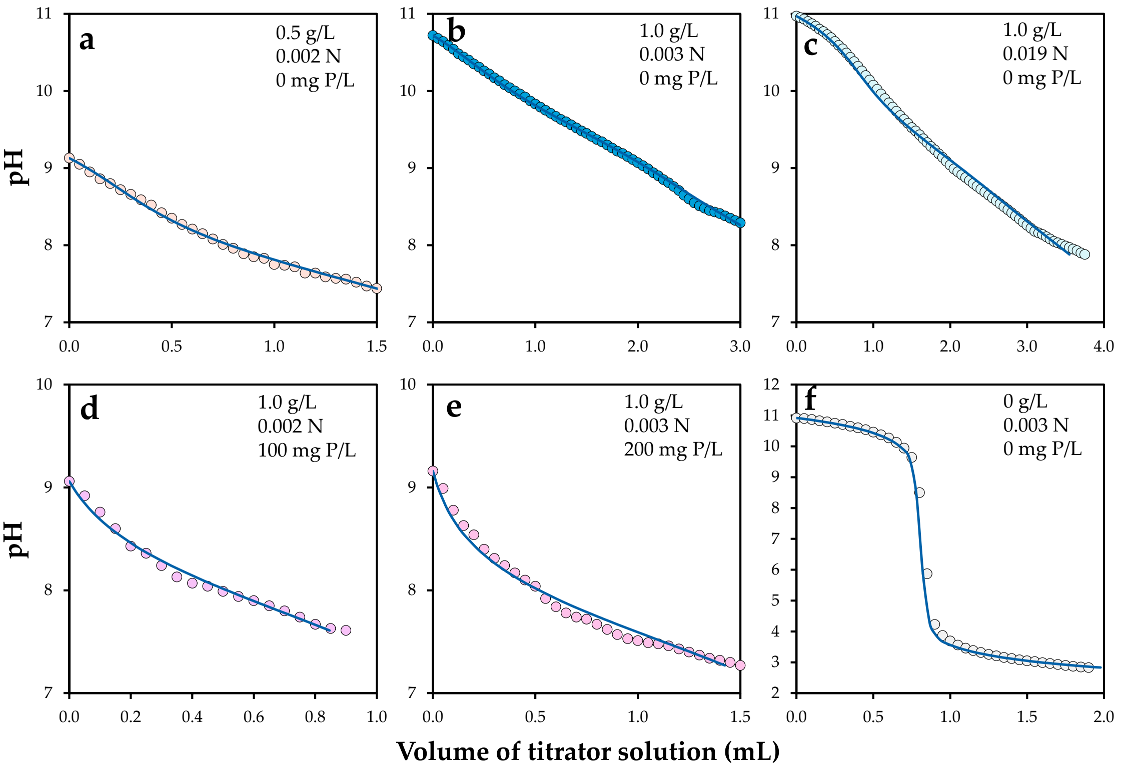

Figure S1. Experimental and Protofit-simulated titration curves of *Posidonia* Ash under different chemical conditions: (a –c) before adsorption, (d – e) after adsorption and (f) background electrolyte.

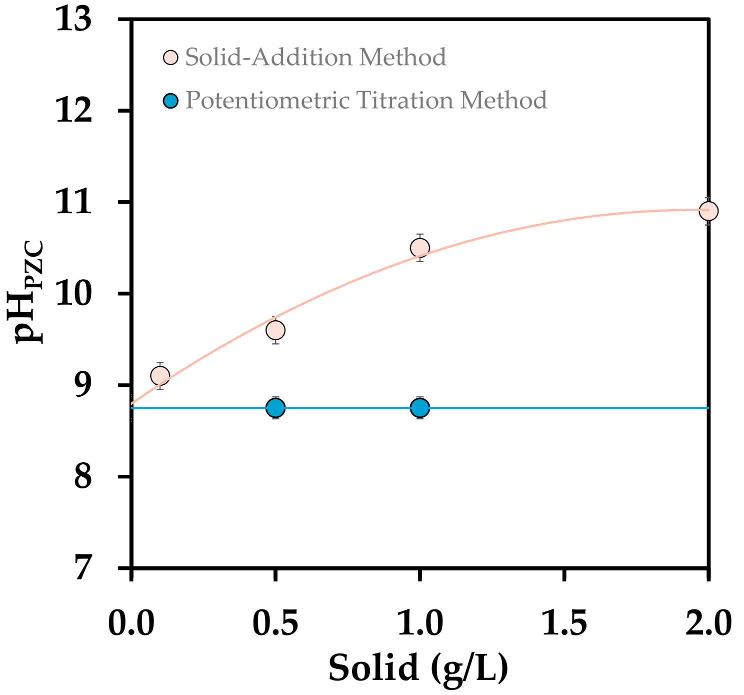

Figure S2. Zero Charge Point as a function of solid dose for different determination methods. Error bars represent the standard error of the mean.

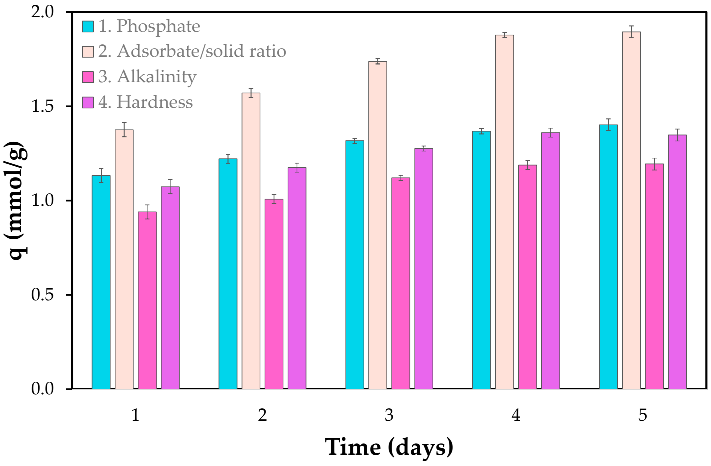

Chemical condition

**Variable**		**Sample**			
		**1**	**2**	**3**	**4**
**Phosphate**	*mg/L*	*50*	*100*	*50*	*50*
**Solid dose**	*g/L*	*1*	*0.5*	*0.5*	*1*
**pH**		*8.7-9.3*	*7.8-8.1*	*9.6-10.2*	*8.0-8.3*
**Alkalinity***	*mg/L*	*10*	*10*	*300*	*10*
**Hardness***	*mg/L*	*0*	*0*	*0*	*300*

Figure S3. Effect of contact time on phosphate adsorption capacity for different chemical conditions: presence of phosphate (1), high adsorbate/solid ratio (2), alkalinity (3) and water hardness (4). Alkalinity and water hardness expressed as $CaCO_3$. Error bars represent the standard error of the mean.

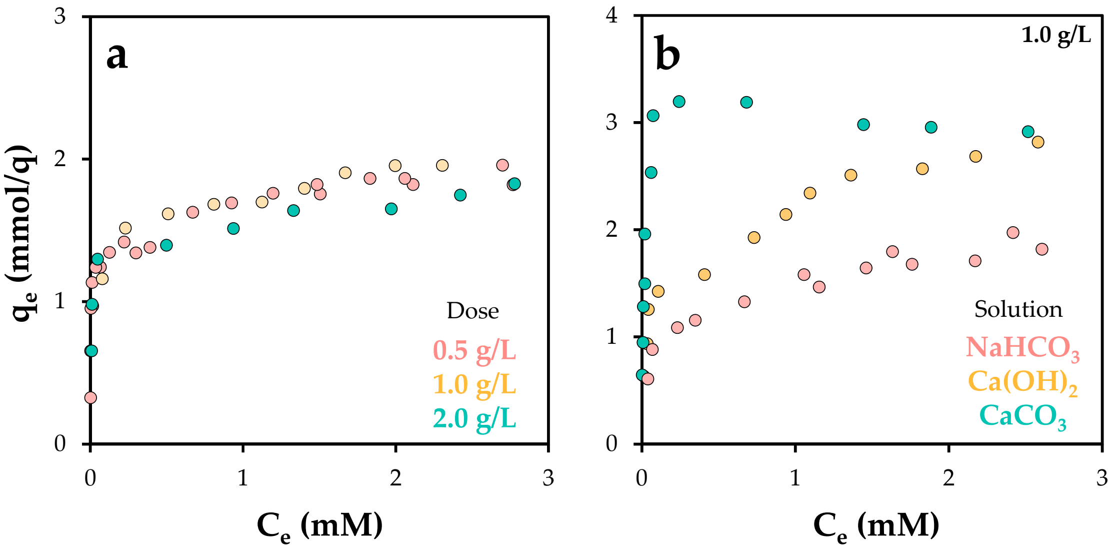

Figure S4. Experimental phosphorus removal capacity at equilibrium as a function of equilibrium dissolved phosphorus concentration, under varying chemical conditions: (a) phosphorus and POA dose changes and (b) different solution conditions. The standard error of the mean ranged from 0.008 to 0.053.

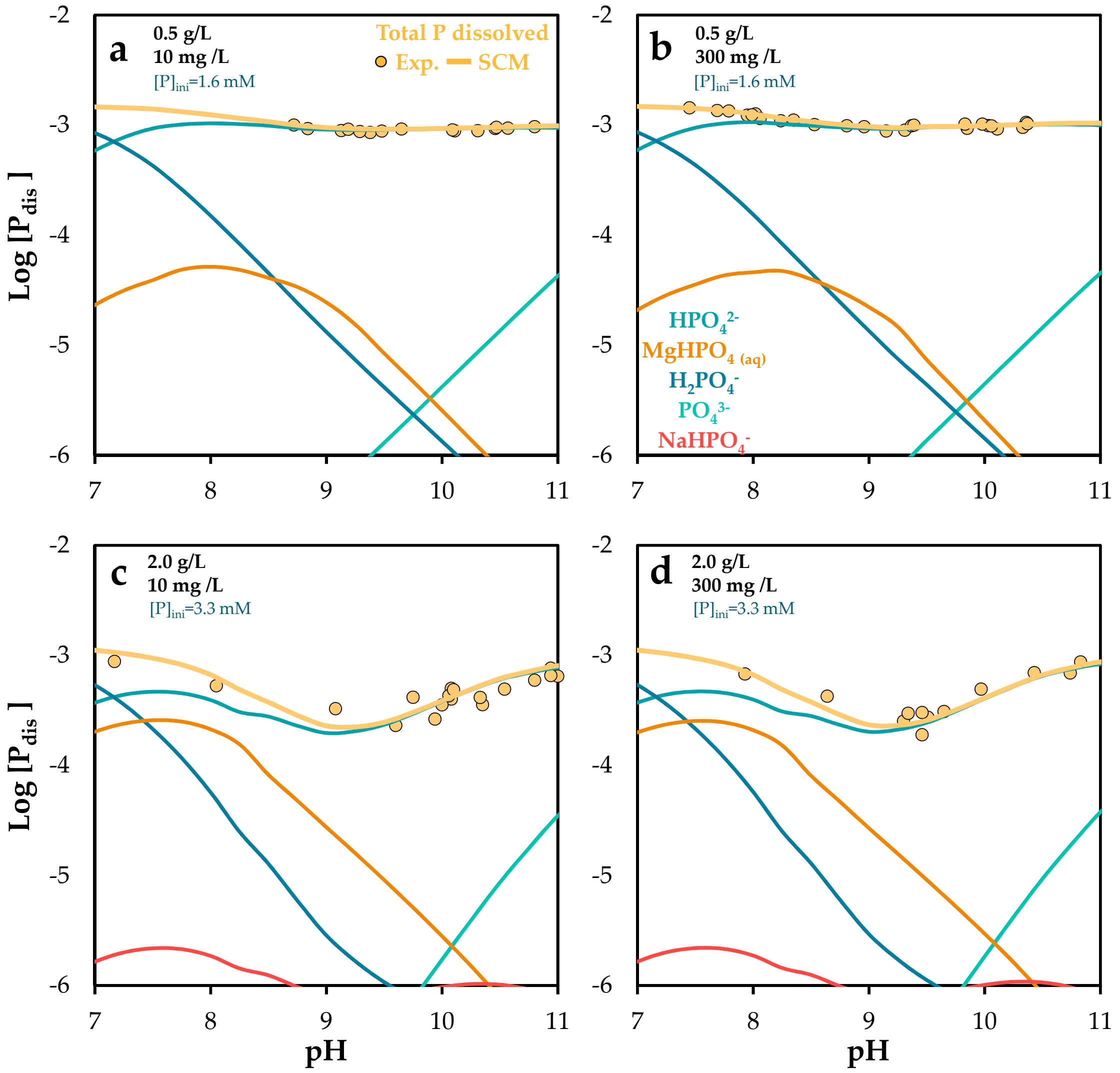

Figure S5. Experimental and Visual MINTEQ-simulated phosphorus dissolved and aqueous phosphorus speciation as a function of pH, at solid doses of (a, b) 0.5 and (c, d) 2.0 g/L, and alkalinity of (a, c) 10 mg/L and (b, d) 300 mg/L as $CaCO_3$. The standard error of the mean ranged from 0.012 to 0.043.

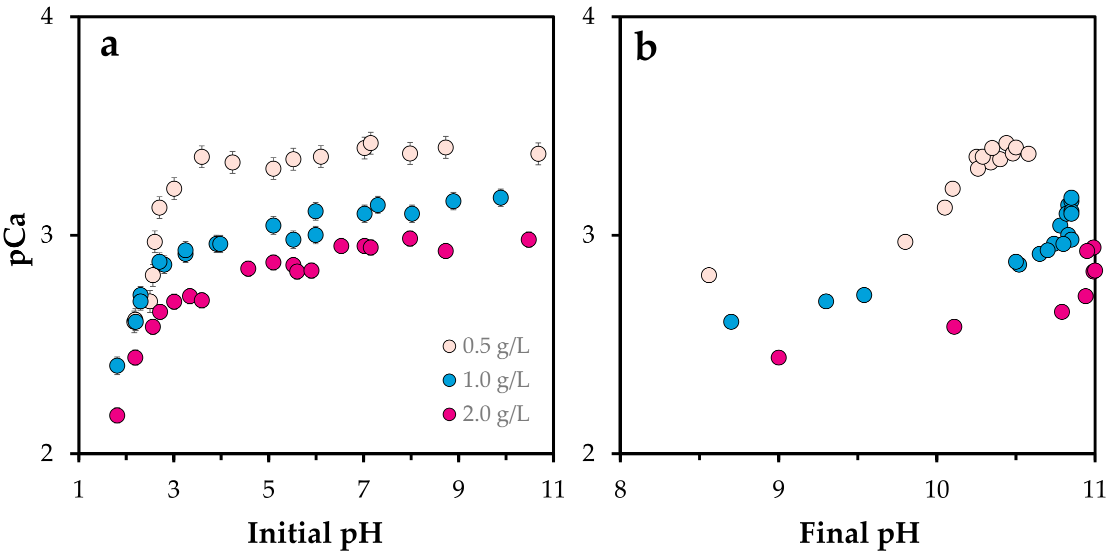

Figure S6. Calcium released by POA adsorbent at different doses as a function of (a) initial and (b) final system pH. The standard error of the mean ranged from 0.016 to 0.049.

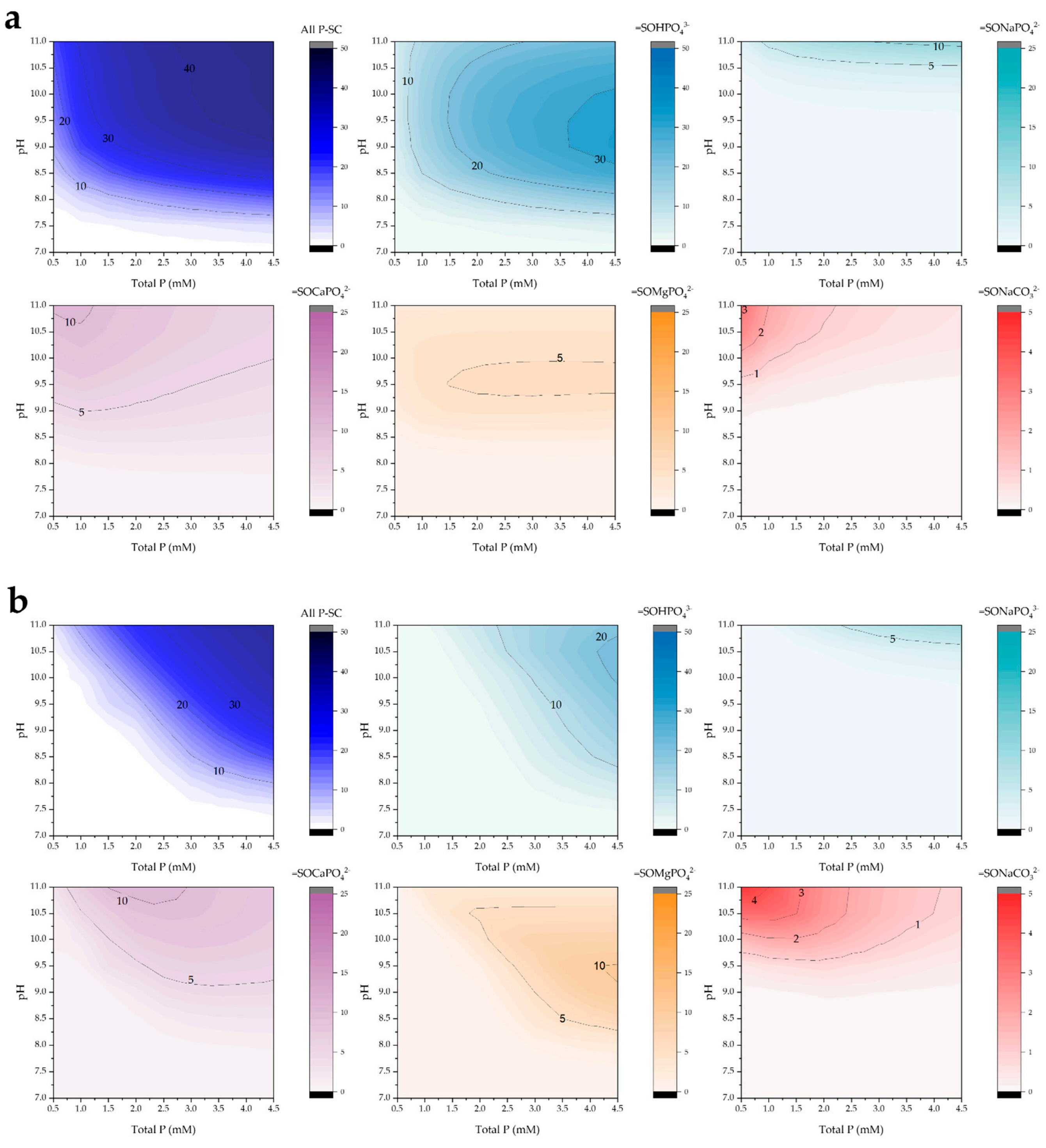

Figure S7. Simulated surface complex predominance (mg/g) as a function of pH and total phosphorus under alkalinity conditions (450 mg/L) for POA doses of (a) 0.5 and (b) 2.0 g/L.
